# A Dual‐Target Recognition System Based on Acid‐Degradable Ni‐MOF and Aptamer Guidance for Precise Tumor Diagnosis and Combined Therapy

**DOI:** 10.1002/advs.202512838

**Published:** 2025-09-24

**Authors:** Jing Xu, Hanxiao Chen, Yifang Tao, Hong Wang, Zhenlong Wang, Yuquan Xue, Delai Fu, Huan Pang, Li Xue

**Affiliations:** ^1^ Department of Urology The Second Affiliated Hospital of Xi'an Jiaotong University Xi'an 710004 China; ^2^ School of Chemistry and Chemical Engineering Yangzhou University Yangzhou 225009 China

**Keywords:** cancer theranostics, dual‐targeting recognition, machine learning

## Abstract

Inherent heterogeneity of tumors significantly limits the therapeutic efficacy of existing cancer treatment systems. This work proposes a dual‐targeting theranostic system based on acid‐responsive cleavage of a metal‐organic framework (MOF). By functionalizing the MOF with dual aptamers that exhibit strict base complementarity to cancer cell biomarkers, the system achieves synergistic passive and active targeting, significantly improving the recognition accuracy for cancer cells. Simultaneously, leveraging the glucose‐dependent metabolic features of tumor cells, the system efficiently catalyzes the generation of hydroxyl radicals (·OH) from glucose, thereby activating chemodynamic therapy. Furthermore, under infrared light irradiation, nickel (Ni) atoms doped within the MOF generate a photothermal effect, further enhancing the inactivation of cancer cells. Both in vitro and in vivo experiments confirm the high efficiency of this system for diagnosis and therapy. The photothermal effect of the MOF material is validated using density functional theory (DFT) calculations, and the therapeutic efficacy is evaluated using a machine learning‐based approach, further demonstrating the system's potential for in vivo therapeutic application. This study provides a novel strategy for precise cancer diagnosis and therapy, offering promising potential to overcome the limitations of existing systems and provide new avenues for cancer theranostics.

## Graphical abstract

This work reports a Ni‐doped ZIF‐8 nanoplatform co‐encapsulating glucose oxidase and horseradish peroxidase, enabling a cascade catalytic reaction that produces hydroxyl radicals from endogenous glucose and hydrogen peroxide. Upon near‐infrared irradiation, the system simultaneously achieves photothermal and chemodynamic therapy, offering an effective synergistic strategy for tumor eradication.

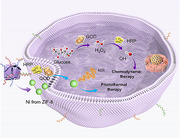

## 1 Introduction

Cancer is one of the major diseases posing a serious threat to human health worldwide, and its high incidence and mortality rates have garnered widespread attention.^[^ ^1^, ^2^, ^3^ ^]^ The heterogeneity of cancer cells and the complexity of the tumor microenvironment have gradually emerged as key factors constraining the effectiveness of cancer diagnosis and therapy.^[^ ^4^, ^5^ ^]^ Cancer cell heterogeneity results in insufficient sensitivity in early diagnosis and low treatment response rates. Meanwhile, the unique characteristics of the tumor microenvironment further limit the efficacy of single‐modality therapies.

As critical biomarkers of cancer cells, microRNAs (miRNAs) have attracted extensive attention due to their pivotal regulatory roles in tumor initiation, progression, and metastasis.^[^ ^6^, ^7^, ^8^, ^9^ ^]^ miRNAs exhibit distinct expression profiles in cancer cells and possess highly conserved sequences, making them ideal targets for precise molecular recognition. For example, miRNA‐221 and miRNA‐155 are significantly upregulated in various cancer types, including prostate cancer, and are closely associated with enhanced cell proliferation, increased invasiveness, metastatic potential, and treatment resistance. Thus, they are considered important indicators for the early detection and risk stratification of prostate cancer.^[^ ^10^ ^]^ Aptamers are short oligonucleotide sequences with high affinity and specificity, capable of binding to target biomolecules through strict base pairing.^[^ ^11^, ^12^, ^13^, ^14^ ^]^ This base‐complementary strategy endows aptamers with excellent specificity, allowing them to accurately identify target molecules even in complex biological environments. By functionalizing dual aptamers on the surface of MOF, the system can achieve precise recognition and targeting of miRNA‐221 and miRNA‐155 via complementary base pairing. This strategy enables the active accumulation of MOF nanoclusters in the tumor microenvironment, thereby significantly enhancing the accuracy and efficiency of cancer diagnosis and therapy.

In the tumor microenvironment, the metabolic characteristics of cancer cells are particularly prominent, among which the Warburg effect is one of the most notable manifestations.^[^ ^15^, ^16^, ^17^ ^]^ Tumor cells produce large amounts of lactic acid through glycolysis, leading to acidification of the microenvironment, while simultaneously releasing substantial amounts of glucose. This unique metabolic pattern not only provides energy to support tumor growth and proliferation but also offers new strategies and potential targets for cancer diagnosis and therapy. MOFs, with their tunable structures, diverse functions, and good biocompatibility, have attracted increasing attention in the field of tumor theranostics in recent years.^[^ ^18^, ^19^, ^20^ ^]^ Among them, ZIF‐8, a typical MOF material, exhibits excellent pH responsiveness and can readily dissociate in the weakly acidic tumor environment, facilitating the release of drugs or functional molecules. In our previous study, dual aptamers were functionalized on the surface of ZIF‐8, enabling precise targeting and recognition of cancer cells through complementary base pairing with miRNA‐221 and miRNA‐155.

As an emerging cancer treatment strategy, chemodynamic therapy (CDT) has gained wide attention due to its high efficiency and low toxicity.^[^ ^21^, ^22^, ^23^ ^]^ CDT relies on in situ catalytic reactions within tumor tissues to generate highly oxidative ·OH, thereby achieving effective cancer cell killing. However, the therapeutic efficacy of CDT alone remains limited due to substrate availability and insufficient oxidative activity. Therefore, developing therapeutic modalities that can synergize with CDT is of great significance. Photothermal therapy (PTT) is a non‐invasive treatment approach that utilizes photothermal conversion materials to convert light energy into heat under near‐infrared (NIR) irradiation, selectively ablating tumor tissues. Some nanomaterials exhibit high efficiency, low toxicity, and tissue penetration capability.^[^ ^24^, ^25^, ^26^ ^]^ Although ZIF‐8 possesses inherent photothermal effects, doping with nickel ions (Ni^2+^) can significantly enhance its photothermal conversion efficiency, enabling rapid temperature elevation under NIR irradiation and effective ablation of tumor tissues. Therefore, Ni‐doped ZIF‐8 not only retains its pH‐responsive release characteristics in the acidic tumor microenvironment but also integrates the synergistic therapeutic potential of PTT and CDT, providing strong support for the construction of multifunctional platforms for precise tumor theranostics.

**Scheme 1:**
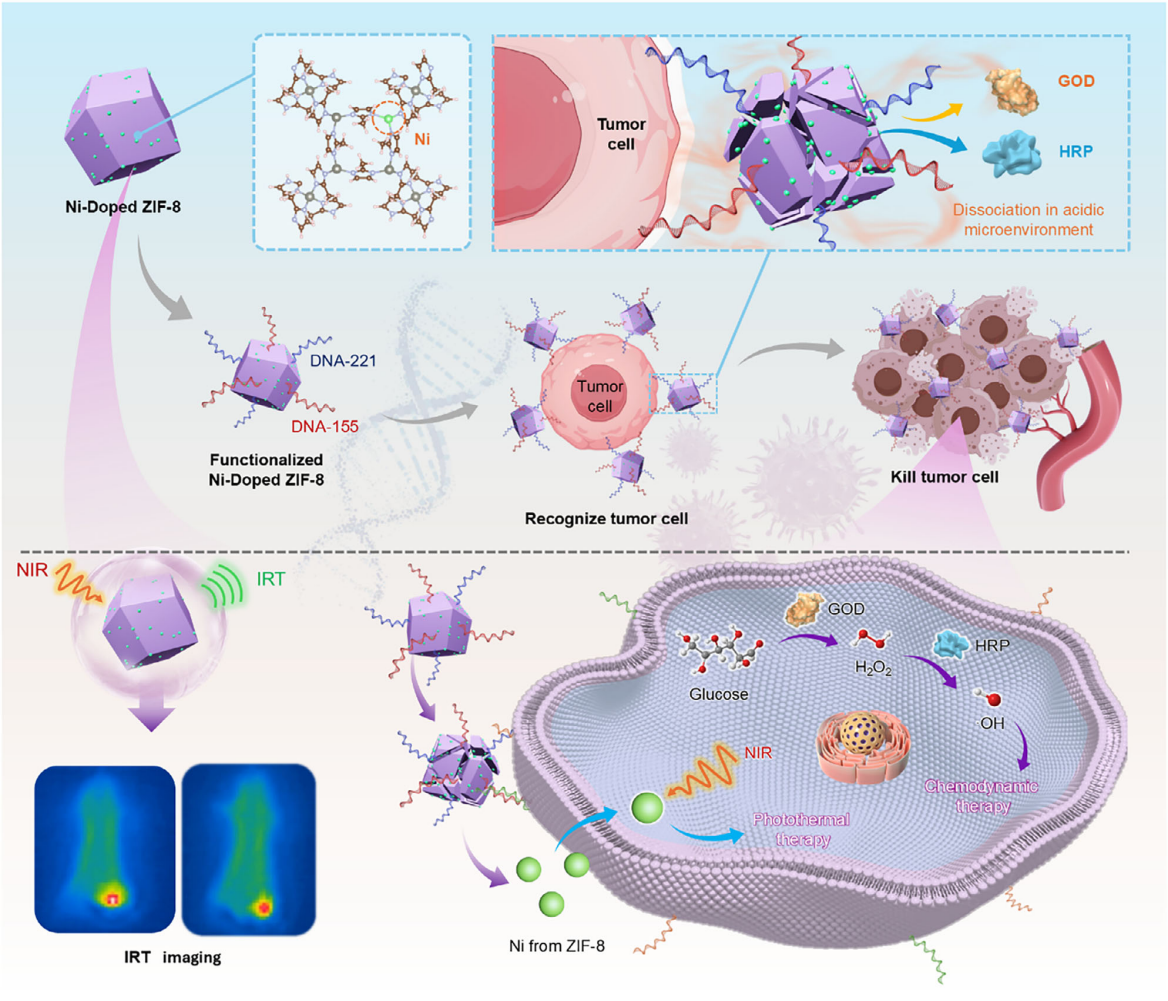
Construction of the Ni‐doped ZIF‐8@GOD&HRP functionalized nanoplatform and its mechanism for tumor cell recognition and therapy.

Herein, two biological enzymes, glucose oxidase (GOD) and horseradish peroxidase (HRP), were encapsulated within Ni‐doped ZIF‐8 nanoparticles possessing intrinsic crystal cavities, and the surface was further modified with aptamer DNA. The resulting composite nanoparticles (DNA@Ni‐ZIF‐8@GOD&HRP) exhibit multiple functional properties, including acid responsiveness and aptamer‐mediated specific recognition. Under the acidic tumor microenvironment, the nanoparticles undergo degradation and release the encapsulated enzymes, which exhibit dual enzymatic activities of GOD and HRP. These enzymes catalyze the conversion of endogenous H_2_O_2_ into cytotoxic ·OH, while simultaneously catalyzing the oxidation of glucose, abundant in the tumor microenvironment, to generate additional H_2_O_2_. This process not only compensates for the insufficient endogenous H_2_O_2_ levels in tumor cells but also enables a continuous ·OH release via a Fenton‐like reaction, thereby achieving specific tumor cell killing through CDT. More importantly, the nanoparticles also serve as efficient photothermal conversion agents, generating pronounced photothermal effects under NIR laser irradiation. This facilitates a synergistic therapeutic approach combining PTT and CDT. Both in in vitro test under simulated conditions and in vivo antitumor experiments confirmed that this nanoparticle‐based dual‐mode detection and therapeutic strategy effectively targets tumor tissues and exhibits excellent antitumor performance, offering promising potential for future applications in multimodal cancer diagnosis and therapy. As illustrated in **Scheme** **1**, the detailed mechanism is illustrated below. The Ni‐doped ZIF‐8 nanomaterial is functionalized by conjugating specific probes targeting miRNA‐155 and miRNA‐221, enabling selective recognition of tumor cells. In the acidic tumor microenvironment, the nanoplatform dissociates and releases the loaded GOD and HRP, which generate ·OH through a cascade enzymatic reaction, thereby achieving efficient tumor cell killing. Meanwhile, the incorporated Ni endows the system with photothermal conversion capability, inducing a photothermal effect under NIR irradiation to further enhance the therapeutic outcome. In addition, the platform employs near‐infrared excitation combined with Infrared Thermography (IRT) imaging to provide visual guidance for precise therapy.

## 2 Results and Discussion

### 2.1 Structural Characterization and Performance Evaluation

**Figure 1:**
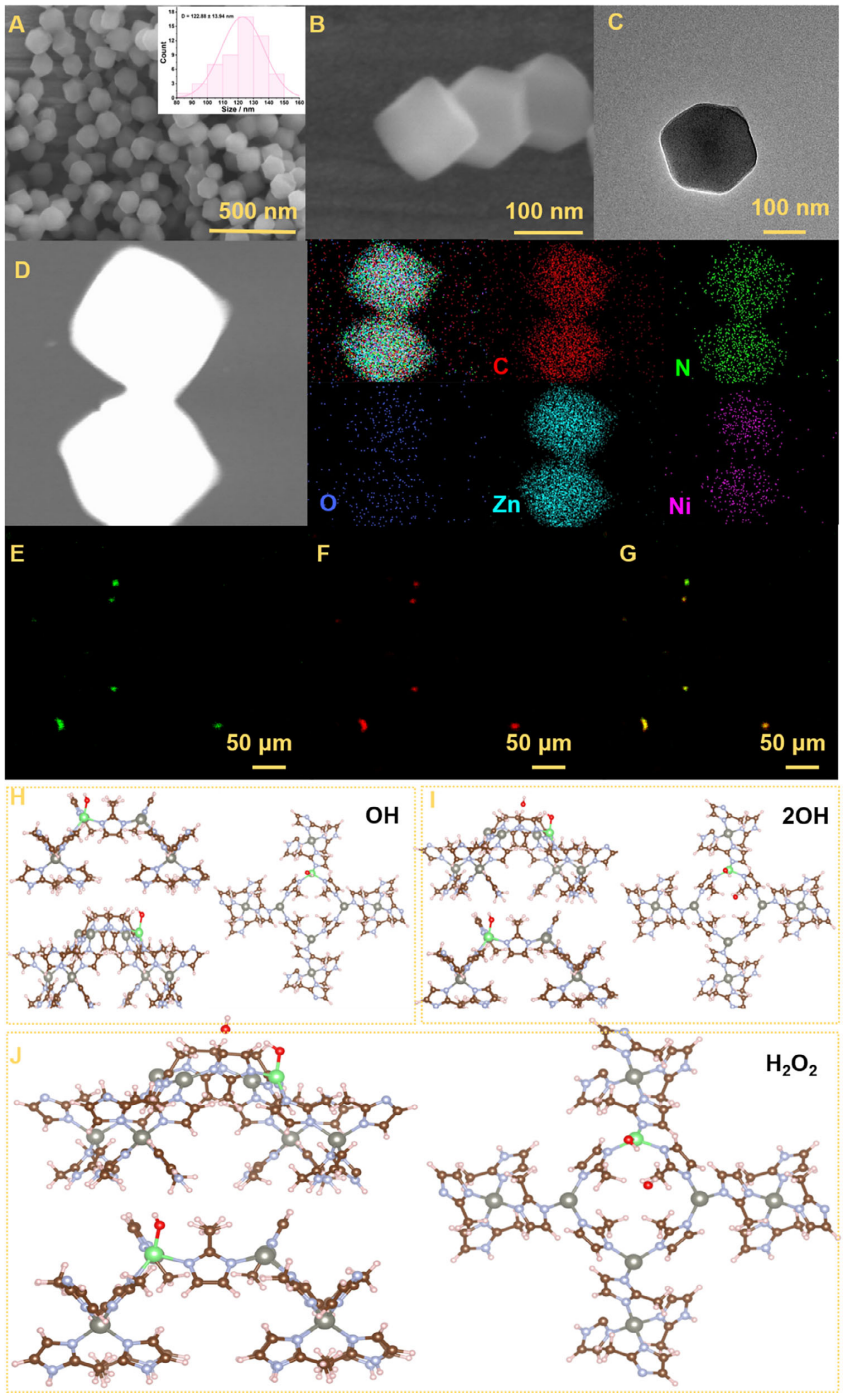
A) SEM image of Ni‐ZIF‐8@GOD&HRP, with the inset showing the size distribution of the nanomaterial under dry conditions as measured by scanning electron microscopy (SEM), B–D) TEM images and corresponding EDX mapping of Ni‐ZIF‐8@GOD&HRP, E–G) Confocal laser scanning microscopy images of dye‐labeled materials, H) Simulation of H_2_O_2_ adsorption and reaction on the surface of Ni‐ZIF‐8, I) Simulation of 2OH adsorption and reaction on the surface of Ni‐ZIF‐8, J) Simulation of OH adsorption and reaction on the surface of Ni‐ZIF‐8.

The morphology of the material is a critical factor influencing enzyme encapsulation efficiency and functional performance, including catalytic activity and stability. Therefore, the morphology of Ni‐ZIF‐8@GOD&HRP was characterized using transmission electron microscopy (TEM) and scanning electron microscopy (SEM) (**Figure** **1**A–C). Uniform dodecahedral crystals with an average size of ≈100 nm were observed. Furthermore, TEM‐based elemental mapping (Figure 1D; Figure S1, Supporting Information) clearly confirmed the coexistence of carbon (C), nitrogen (N), oxygen (O), zinc (Zn), and nickel (Ni) within the crystals. To verify the successful encapsulation of both enzymes, GOD and HRP were labeled with fluorescein isothiocyanate (FITC, green) and rhodamine B (RhB, red), respectively. The super‐resolution confocal images showed strong fluorescence signals from the material (Figure 1E–G), confirming the successful synthesis of Ni‐ZIF‐8@GOD&HRP. The morphology and size distribution of Ni‐ZIF‐8@GOD&HRP nanoparticles were further characterized by SEM and dynamic light scattering (DLS). SEM images showed a uniform dry diameter of 122.88 ± 13.94 nm (Figure 1A). When dispersed in simulated blood plasma, DLS measurements indicated a hydrodynamic size of ≈375.9 nm with a low polydispersity index (PDI = 0.278), demonstrating good dispersion without obvious aggregation (Figure S2A,B, Supporting Information). To gain deeper insight into the surface adsorption and catalytic mechanisms, DFT simulations were carried out. Figure 1H–J illustrates the adsorption and reaction configurations of OH, 2OH, and H_2_O_2_ on the Ni‐ZIF‐8 surface, providing a theoretical basis for subsequent catalytic analysis of Ni‐ZIF‐8.

To evaluate the PTT potential of Ni‐ZIF‐8, its photothermal properties were systematically investigated. As shown in Figure S3 (Supporting Information), the photothermal performance of Ni‐ZIF‐8 was evaluated under 808 nm irradiation at a power density of 1.0 W·cm^−2^. Under 808 nm laser irradiation, Ni‐ZIF‐8 exhibited significant photothermal conversion efficiency (Figure S3A, Supporting Information). Specifically, the cooling curve obtained from heating‐cooling cycles of Ni‐ZIF‐8 was fitted, and the slope was used to calculate the photothermal conversion efficiency based on the method reported in the literature,^[^ ^27^ ^]^ yielding a value of 34.22%. Additionally, four cycles of heating‐cooling experiments were conducted. After four cycles, the photothermal performance of Ni‐ZIF‐8 showed no noticeable change (Figure S3B, Supporting Information), indicating excellent thermal stability and photothermal conversion capability. To further investigate the effect of the acidic tumor microenvironment on the photothermal performance of Ni‐ZIF‐8@GOD&HRP, systematic tests were conducted under pH 5.0 conditions (Figure S3C,D, Supporting Information). The results showed that, compared with pH 7.4, the photothermal conversion efficiency at pH 5.0 decreased to 23.27%, with a maximum temperature of ≈51 °C. After four laser on/off cycles, the photothermal performance under pH 5.0 exhibited a certain degree of attenuation, which may be related to partial structural degradation or changes in surface properties. Despite the slight reduction in efficiency and temperature, the heating level remained sufficient to achieve effective photothermal therapy within a short period, indicating that the material retains good application potential in acidic tumor microenvironments.

**Figure 2:**
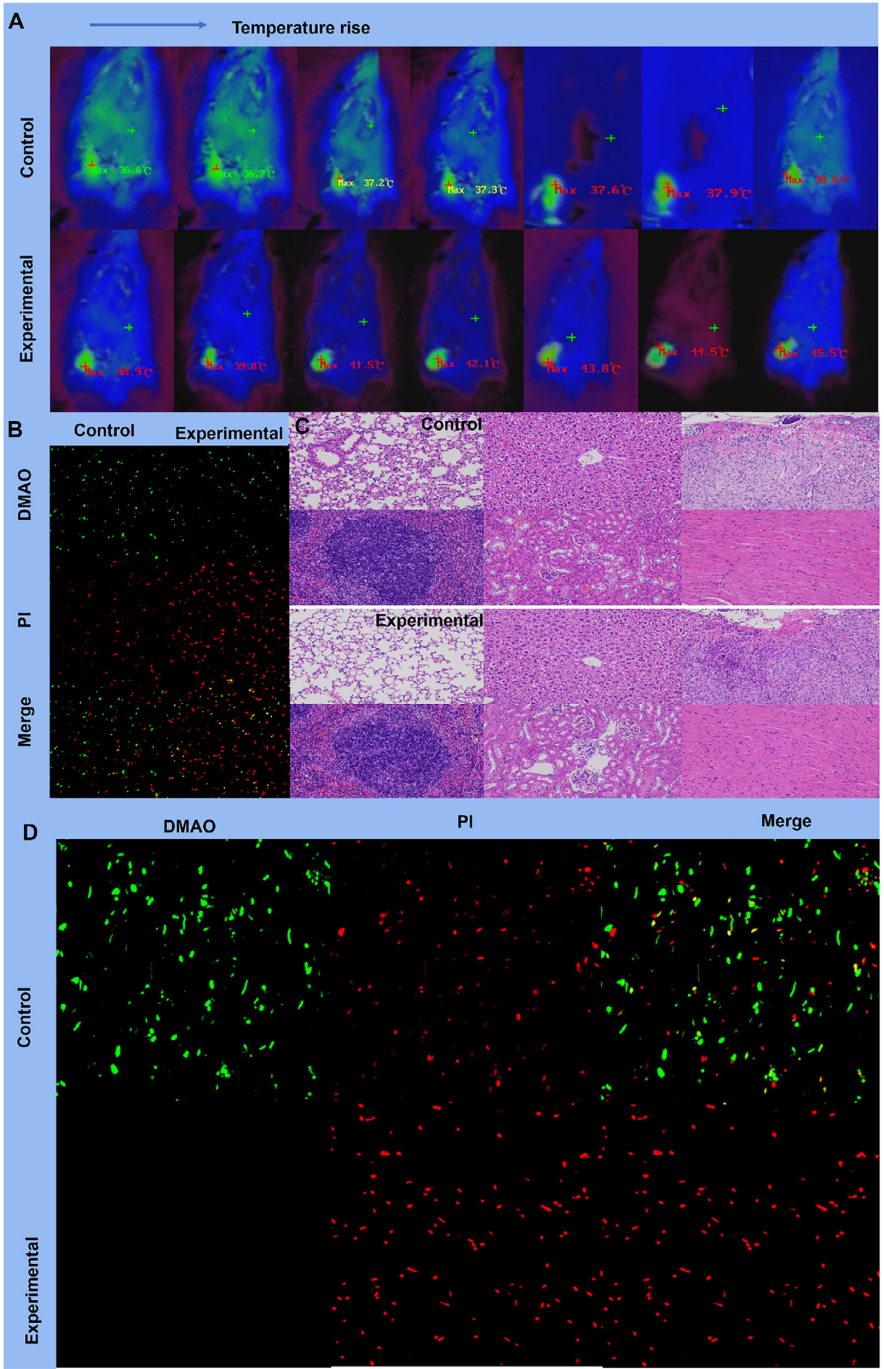
A) Infrared thermal images showing the temperature rise in mice from the control and treated groups. B) Live/dead bacterial staining (DMAO/PI). C) Hematoxylin‐eosin (H&E) staining of major organs from mice in the control and treated groups, D) Confocal microscopy images of antibacterial performance under different treatment conditions (Scale bar, 50 µm).

For in vivo drug delivery applications, intrinsic antibacterial activity is a desirable property. To evaluate the in vivo antibacterial effect of the nanoparticle‐mediated photothermal therapy, infrared thermal imaging was first performed. Upon laser irradiation, the treated group exhibited a continuous increase in local temperature, whereas the control group showed only slight changes, indicating efficient photothermal conversion and therapeutic heat accumulation (**Figure** **2**A). Furthermore, live/dead bacterial staining of homogenized infected tissues revealed a marked reduction in viable bacteria and a predominance of dead bacteria in the treated group, confirming the strong antibacterial activity of the therapy (Figure 2B). Importantly, hematoxylin‐eosin (H&E) staining of major organs showed no evident inflammatory cell infiltration or tissue damage in either group, suggesting that the treatment did not induce significant systemic toxicity (Figure 2C). Collectively, these findings verify that the nanoparticle‐based photothermal therapy can achieve good biosafety while exhibiting favorable antibacterial performance. In addition, in vitro antibacterial performance tests were conducted. Compared with the control group (irradiated for 25 min only), the bacterial survival rate decreased significantly after 25 min of infrared irradiation (Figure 2D; Figure S4, Supporting Information). The remarkable photothermal antibacterial effect of Ni‐ZIF‐8 further confirmed its excellent photothermal conversion efficiency and antibacterial properties. Meanwhile, as shown in Figure S5 (Supporting Information), enzyme activity was measured after incubating the enzymes at 50 °C for 10 min to simulate the thermal effect during PTT. The results indicate that both enzymes maintained good activity under these conditions.

### 2.2 Materials Structure and Properties Analysis

**Figure 3:**
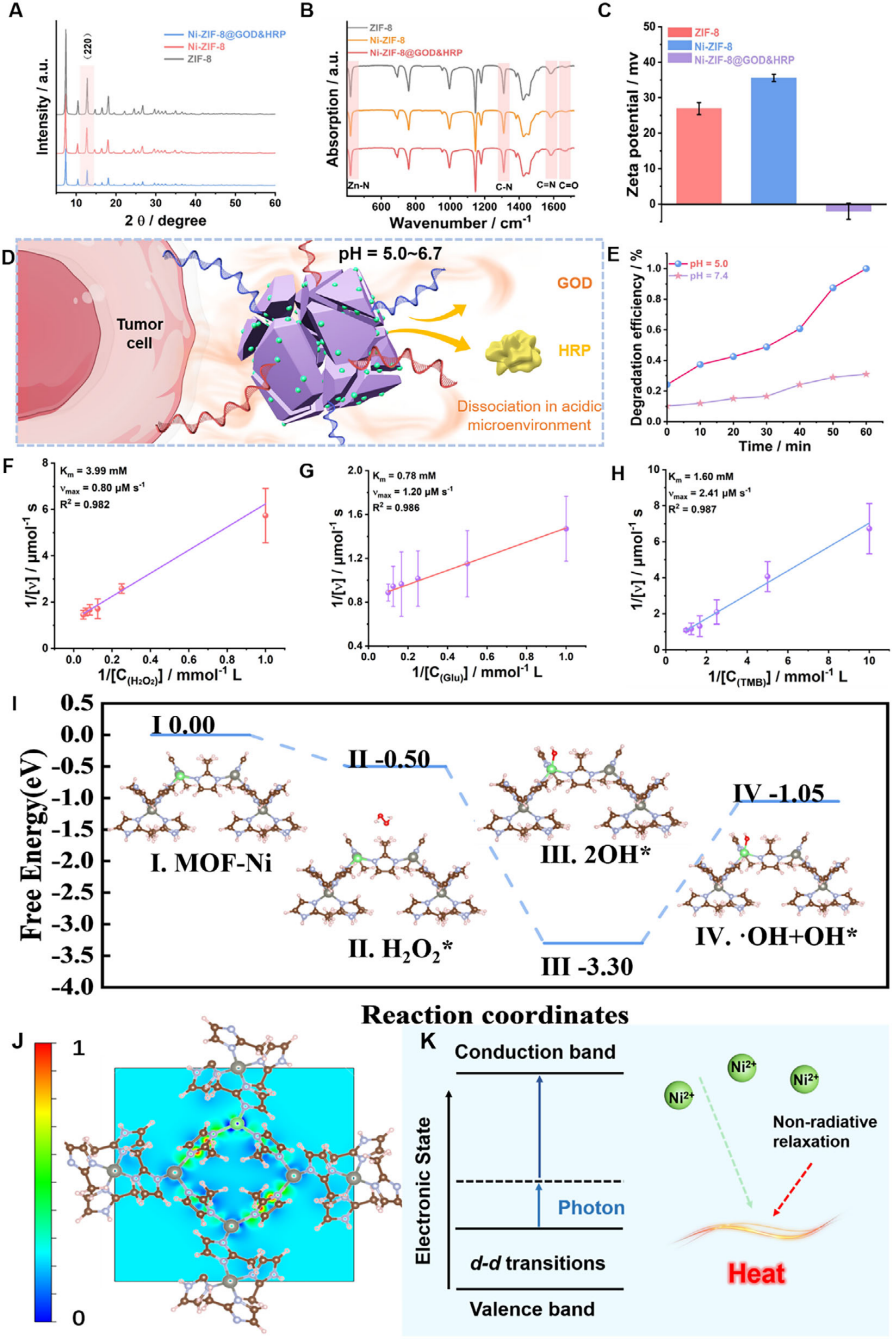
A–C) XRD patterns, FTIR spectra, and zeta potential measurements of ZIF‐8, Ni‐ZIF‐8, and Ni‐ZIF‐8@GOD&HRP, D) Schematic illustration of the degradation mechanism of Ni‐ZIF‐8@GOD&HRP under the tumor microenvironment, E) Normalized degradation efficiency of Ni‐ZIF‐8@GOD&HRP at different pH values, F–H) Lineweaver‐Burk plots (*n* = 3) showing the relationship between catalytic activity and substrate concentration (H_2_O_2_, glucose, and TMB), I) Free energy diagram for H_2_O_2_ decomposition at the Ni active site, J) Schematic illustration of the role of Ni^2+^ in the photothermal conversion process within ZIF‐8, K) Ni‐ZIF‐8 electronic localization function (ELF) distribution diagram.

As shown in the Raman spectrum (Figure S6, Supporting Information), a weak vibrational peak corresponding to Ni‐N bonding appears at ≈500 cm^−1^ in Ni‐ZIF‐8, in contrast to ZIF‐8, confirming the successful incorporation of nickel and its influence on the molecular vibrational characteristics of the material. Meanwhile, the X‐ray diffraction (XRD) results indicate that the crystallinity of Ni‐ZIF‐8@GOD&HRP is significantly reduced due to the presence of GOD and HRP; however, the peak positions remain consistent with those of Ni‐ZIF‐8 and ZIF‐8 (**Figure** **3**A), suggesting that the overall crystalline structure is retained. X‐ray photoelectron spectroscopy (XPS), including the survey spectrum (Figure S7A, Supporting Information) and high‐resolution spectra (Figure S7B–F, Supporting Information), was employed to determine the chemical states and relative elemental compositions of the Ni‐ZIF‐8@GOD&HRP composite. The high‐resolution spectra (Figure S7B–F, Supporting Information) reveal binding energy peaks corresponding to C 1s, N 1s, O 1s, Zn 2p, and Ni 2p, confirming the presence of C─N, C═O, and Ni─N chemical bonds and providing insights into the electronic environment on the material's surface. In addition, the Fourier‐transform infrared (FTIR) spectrum exhibits characteristic vibrational peaks in the range of 1600–1700 cm^−1^, which are primarily attributed to the C═O stretching vibrations of the amide groups present in GOD and HRP (Figure 3B). Furthermore, the Zeta potential increased from +26.9 mV for ZIF‐8 to +35.6 mV upon Ni doping, indicating enhanced surface positivity. A substantial decrease in Zeta potential from +35.6 mV (Ni‐ZIF‐8) to −2.0 mV (Ni‐ZIF‐8@GOD&HRP) further confirms successful enzyme encapsulation (Figure 3C), as the negatively charged GOD and HRP partially neutralize the surface charges of the positively charged Ni‐ZIF‐8 framework. Collectively, these characterization results provide strong evidence for the successful synthesis of the Ni‐ZIF‐8@GOD&HRP composite.

The enzyme loading efficiency has a direct impact on the therapeutic efficacy. Figure S8A,B (Supporting Information) show the fluorescence intensities of the supernatant and sequential washing solutions collected during the in situ encapsulation of HRP and GOD, respectively. Enzyme concentrations were determined from the calibration curves (Figure S9A–D, Supporting Information), yielding loading contents of 2.6 wt.% (HRP) and 3.6 wt.% (GOD). To further evaluate the effect of enzyme encapsulation on the porosity of Ni‐doped ZIF‐8 nanoparticles, Brunauer–Emmett–Teller (BET) surface area and pore size distribution measurements were conducted. The results showed that the surface area slightly decreased from 1294.8 m^2^ g^−1^ for pristine Ni‐ZIF‐8 to 1276.5 m^2^ g^−1^ after enzyme encapsulation, while the average pore size decreased from 2.542 to 2.348 nm (Figure S10A–D, Supporting Information). These moderate reductions can be attributed to the successful encapsulation of GOD and HRP within the framework pores, partially occupying the intrinsic channels without significantly compromising the overall porous structure. Acid‐responsive degradation is a key feature of ZIF‐8. As illustrated in Figure 3D, in the acidic tumor microenvironment, protons (H^+^) bind to the nitrogen atoms of the imidazole ligands, weakening or breaking the coordination bonds between the ligands and Zn^2+^. Upon bond dissociation, Zn^2+^ may further combine with anions in the surrounding solution to form soluble salts, which disrupt the structural integrity of the framework, ultimately leading to the decomposition of ZIF‐8 and the controlled release of GOD and HRP. The degradation profile of Ni‐ZIF‐8@GOD&HRP (Figure 3E; Figure S8C,D, Supporting Information) demonstrates that the nanoplatform degrades significantly faster under acidic conditions (pH = 5.0) compared to neutral conditions (pH = 7.4), confirming the enhanced degradation behavior under tumor‐relevant acidic environments. The catalytic activity was evaluated by monitoring the reactions of H_2_O_2_ with three chromogenic substrates‐3,3′,5,5′‐tetramethylbenzidine (TMB), methylene blue (MB), and 3,3′‐diaminobenzidine (DAB)‐and measuring their absorbance at the respective characteristic wavelengths. The results revealed that Ni‐ZIF‐8 exhibited higher catalytic activity toward H_2_O_2_ than ZIF‐8, indicating that nickel doping enhanced the intrinsic catalytic capacity of the framework (Figure S11, Supporting Information). Moreover, Ni‐ZIF‐8@GOD&HRP showed the highest catalytic efficiency toward all three substrates, underscoring the synergistic effect of GOD and HRP immobilized within the Ni‐ZIF‐8 framework in boosting catalytic performance. To further investigate the catalytic kinetics of the Ni‐ZIF‐8@GOD&HRP nanoplatform toward H_2_O_2_, the effects of substrate concentrations (H_2_O_2_, glucose, and TMB) and reaction time were systematically analyzed (Figure S12A–F, Supporting Information). The calculated Michaelis‐Menten constants (K_m_) were 3.99 mm for H_2_O_2_, 0.78 mm for glucose, and 1.60 mm for TMB. The maximum reaction rates (V_max_) were 0.80 µm·s^−1^ for H_2_O_2_, 1.20 µm·s^−1^ for glucose, and 2.41 µm·s^−1^ for TMB (Figure 3F–H and *n* = 3), outperforming most reported peroxidase‐mimicking catalysts, indicating excellent catalytic activity of the Ni‐ZIF‐8@GOD&HRP nanocomposite (Table S2, Supporting Information). The free energy diagram presented in Figure 3I illustrates the H_2_O_2_ decomposition pathway on the Ni sites of Ni‐ZIF‐8. The desorption of ·OH was identified as the rate‐determining step. The adsorption energy of H_2_O_2_ was calculated to be −0.5 eV, suggesting thermodynamically favorable adsorption at the Ni sites, and the decomposition of H_2_O_2_ was also found to be thermodynamically spontaneous. In summary, these findings demonstrate that the developed nanomaterial not only effectively targets tumor sites but also exhibits enhanced degradation and catalytic activity in the acidic tumor microenvironment. It catalyzes the decomposition of H_2_O_2_ efficiently to generate abundant ·OH, providing a promising strategy for hydroxyl radical‐mediated tumor therapy. Figure 3J presents the electron localization function (ELF) distribution of the Ni‐ZIF‐8 framework, illustrating the electronic structure modulation induced by Ni doping and its potential contributions to catalytic and photothermal performance. Highly localized electron regions (ELF values close to 1) are conducive to the formation of catalytically active sites, whereas delocalized electron regions (ELF values close to 0) facilitate rapid electron transport throughout the system. This balanced distribution of localized and delocalized electrons not only endows the Ni centers with potential catalytic activity but also enhances overall electron transport efficiency. In terms of photothermal performance, electron delocalization at the Ni centers promotes the rapid migration and separation of photoexcited charge carriers, reduces radiative recombination, and facilitates the conversion of energy into lattice vibrations (heat) via non‐radiative relaxation. Furthermore, the unfilled d‐orbitals of Ni^2+^ can undergo ligand‐field (d‐d) transitions under light irradiation, thereby extending the absorption spectrum into the visible‐near‐infrared region and directly participating in photothermal energy conversion. The synergistic distribution of localized and delocalized electrons enables Ni‐ZIF‐8 to combine efficient electron transport with enhanced photothermal conversion performance (Figure 3K).

### 2.3 Sensor Assembly and Detection

**Figure 4:**
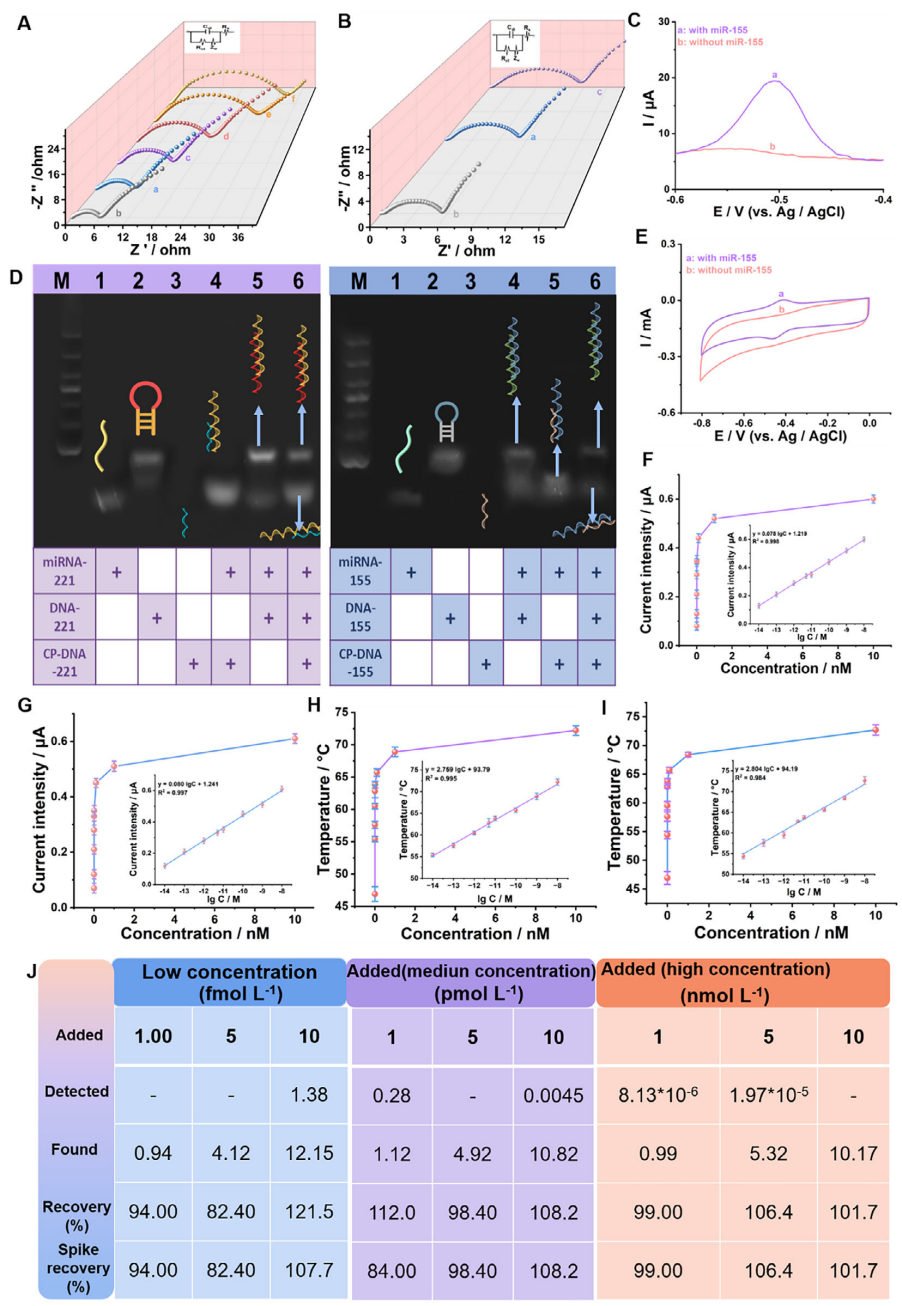
A) EIS diagram: CP (a), Ni‐ZIF‐8/AuNPs/CP (b), DNA‐221, DNA‐155/ Ni‐ZIF‐8/AuNPs/CP (c), MCH/DNA‐221, DNA‐155/Ni‐ZIF‐8/AuNPs/CP (d), miRNA‐221, miRNA‐155/MCH/DNA‐221, DNA‐155/ Ni‐ZIF‐8/AuNPs/CP (e), CP‐DNA‐221‐GOD, CP‐DNA‐155‐GOD/miRNA‐221, miRNA‐155/MCH/DNA‐221, DNA‐155/Ni‐ZIF‐8/AuNPs/CP (f), B) Bio‐cathode: CP (a), Ni‐ZIF‐8/AuNPs/CP (b), BOD/Ni‐ZIF‐8/AuNPs/CP (c), C) DPV curves with and without miRNA‐221 D) Agarose gel electrophoresis diagram, E) CV curves with and without miRNA‐155, F,G) Transient current responses at various concentrations of miRNA‐221/155 and their linear correlation with the logarithm of concentration; H,I) Temperature values measured at different concentrations of miRNA‐221/155 and the corresponding linear relationship with the logarithmic concentration, J) Spike‐and‐recovery assay of miRNA‐221 in serum.

Electrochemical impedance spectroscopy (EIS) was employed to investigate the stepwise assembly process of the bioelectrode. As shown in **Figure** **4**A, the charge transfer resistance (Rct) of the electrode modified with Ni‐ZIF‐8/AuNPs (curve b) was lower than that of the bare CP electrode (curve a), indicating that the Ni‐ZIF‐8/AuNPs composite not only possessed excellent conductivity but also reduced interfacial electron transfer resistance. Subsequent modification with DNA‐221/155 (curve c) led to an increase in Rct, likely due to the introduction of negatively charged DNA molecules. As additional components were introduced, MCH (curve d), miRNAs‐221/155 (curve e), and CP‐DNA‐221‐GOD/CP‐DNA‐155‐GOD (curve f), the Rct values continued to increase as a result of steric hindrance and accumulated surface charge effects. Similarly, in Figure 4B, modification of the Ni‐ZIF‐8/AuNPs electrode with bilirubin oxidase (BOD) significantly increased the resistance due to the prominent steric hindrance associated with the enzyme. The electrochemical activity of the biocathode under various gas environments was evaluated using cyclic voltammetry (CV), as shown in Figure S13A (Supporting Information). A pronounced redox peak current was observed in O_2_‐saturated PBS, compared to N_2_‐ and air‐saturated solutions, indicating efficient oxygen reduction by BOD and successful fabrication of the biocathode. Figures 4C and S13B–D (Supporting Information) demonstrate the distinct oxidation peaks in the DPV and LSV curves only in the presence of the target miRNA‐221 or miRNA‐155, while no such peaks appeared in their absence (curve a), indicating high specificity of the bioanode toward target molecules. Notably, as shown in Figure 4E, a pair of redox peaks attributed to GOD was observed at −0.5 V, consistent with the classical catalytic mechanism of GOD‐mediated glucose oxidation. Moreover, as shown in Figure S14A–D (Supporting Information), the surface morphology of the carbon cloth and chip substrates changed from smooth to rough after modification, indicating that both the material and the probes were successfully immobilized and the surface reactions were effectively completed.

To enhance the specificity of miRNA‐221 and miRNA‐155 detection, a target‐specific bio‐conjugation strategy was designed (Figure S15, Supporting Information). The feasibility of this system was validated by agarose gel electrophoresis (AGE, Figure 4D). Lanes 1–3 correspond to miRNA‐221/155, DNA‐221/155, and CP‐DNA‐221/155 alone, each exhibiting distinct migration bands. Due to their short sequences, the CP‐DNA bands migrated faster and partially moved out of the gel. Upon hybridization of CP‐DNA‐221/155 with miRNA‐221/155 and miRNA‐221/155 with DNA‐211/155, the bands shifted upward and migrated more slowly, confirming successful duplex formation and efficient base pairing. As shown in Figure S16 (Supporting Information), the optimal detection conditions were determined to be a CP‐DNA concentration of 10 nm, a hybridization temperature of 37 °C, and a hybridization time of 60 min, at which the current response reached its maximum. As shown in Figure 4F,G, the peak current exhibited a strong positive correlation with the concentration of miRNA‐221/155, reaching maximum values under optimal conditions. A strong linear relationship was observed between the peak current and the logarithm of miRNA concentrations ranging from 10 fmol L^−1^ to 10 nmol L^−1^. The calibration equations were y = 0.080 log C + 1.241 (R^2^ = 0.997) for miRNA‐221 and y = 0.078 log C + 1.219 (R^2^ = 0.998) for miRNA‐155, with detection limits of 0.15 and 0.14 fmol L^−1^, respectively (*S/N* = 3). For thermal signal detection, TMB was introduced as a chromogenic substrate. Upon incubation with varying concentrations of miRNAs, H_2_O_2_ generated at the bioanode was catalytically converted into oxTMB by Ni‐ZIF‐8@GOD&HRP, which possesses photothermal properties. The corresponding temperature changes under 808 nm laser irradiation were recorded using an infrared thermal imaging camera. As shown in Figure 4H,I, the temperature increased with increasing miRNA concentrations. Within the concentration range of 10 fmol L^−1^ to 10 nmol L^−1^, a strong linear relationship was observed between miRNA concentration and temperature. The regression equations were y = 2.759 log C + 93.79 (R^2^ = 0.995) for miRNA‐221 and y = 2.804 log C + 94.19 (R^2^ = 0.984) for miRNA‐155, with detection limits of 1.34 and 1.42 fmol L^−1^, respectively (*S/N* = 3). Furthermore, the practical applicability of the biosensor was evaluated by spiked recovery experiments in human serum samples. As shown in Figures 4J and S17A (Supporting Information), recovery rates ranged from 82.40% to 108.2% for miRNA‐221 and from 89.00% to 119.0% for miRNA‐155, demonstrating the biosensor's strong potential for real‐sample analysis. In conclusion, the proposed self‐powered biosensor offers dual‐mode (electrochemical and thermal) detection capability for miRNA‐221 and miRNA‐155. As summarized in Figure S17B (Supporting Information), the biosensing platform exhibits outstanding performance in miRNA detection, providing a promising strategy for early and accurate diagnosis of disease biomarkers.

### 2.4 Sensor Performance Analysis

**Figure 5:**
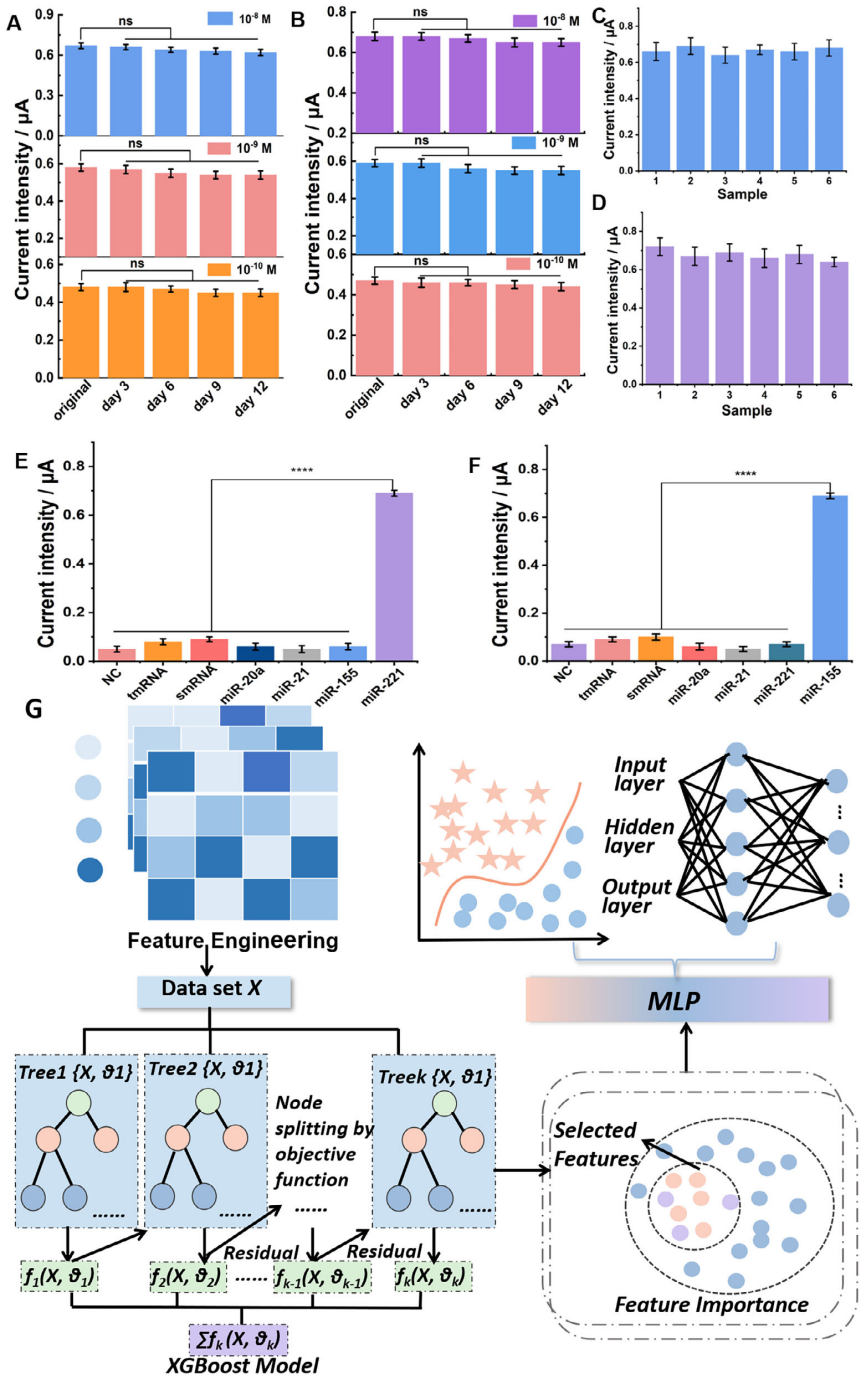
A,B) Stability analysis of miRNA‐221 and miRNA‐155 over time. C,D) Reproducibility of the sensor response for miRNA‐221 and miRNA‐155. E,F) Selectivity of the sensor toward miRNA‐221 and miRNA‐155 against potential interfering sequences, G) Data analysis flowchart.

The stability of the self‐powered biosensor was evaluated by storing the devices at 4  °C for 3, 6, 9, and 12 days. As shown in **Figure** **5**A,B, the instantaneous current responses of the biosensor to target molecules at different concentrations (ranging from 10^−8^ to 10^−10^ mol L^−1^) remained highly consistent over time, with signal retention exceeding 90%, indicating excellent stability. The reproducibility of the biosensor for detecting the two target miRNAs was assessed by performing parallel measurements using six independently fabricated self‐powered biosensors. As shown in Figure 5C,D, the relative standard deviation (RSD) for both miRNA‐221 and miRNA‐155 was ≈5.0%, demonstrating good reproducibility. To further evaluate the selectivity of the biosensor, three non‐target miRNAs (miR‐20a, miR‐21, and miR‐221) and three mismatched sequences based on miR‐155 (smRNA, tmRNA, and NC) were used as interfering agents at a concentration of 1 µmol L^−1^ (Figure 5E,F). Similarly, three unrelated miRNAs (miR‐20a, miR‐21, and miR‐155) and three miR‐221‐derived mismatched sequences (smRNA, tmRNA, and NC) were employed to assess interference for the detection of miR‐221. In both cases, the instantaneous current responses generated by the interfering sequences were significantly lower than those induced by the corresponding target miRNAs (*p* < 0.0001), confirming the high specificity and excellent selectivity of the biosensor. To enhance the accuracy and reliability of miRNA expression analysis, a bioinformatics prediction model was established using deep learning algorithm to forecast changes in miRNA expression. As illustrated in Figure 5G, the pipeline begins with feature extraction using Cartesian difference (CartDiff) and feature engineering (FeatEng). The most informative features are then selected via the XGBoost algorithm. Subsequently, a multi‐layer perceptron (MLP) model integrated with an attention mechanism is defined and trained using K‐fold cross‐validation. The final model outputs predictive results for the expression changes of miRNA‐221 and miRNA‐155.

### 2.5 Deep Learning‐Assisted Detection and In Vivo Validation

**Figure 6:**
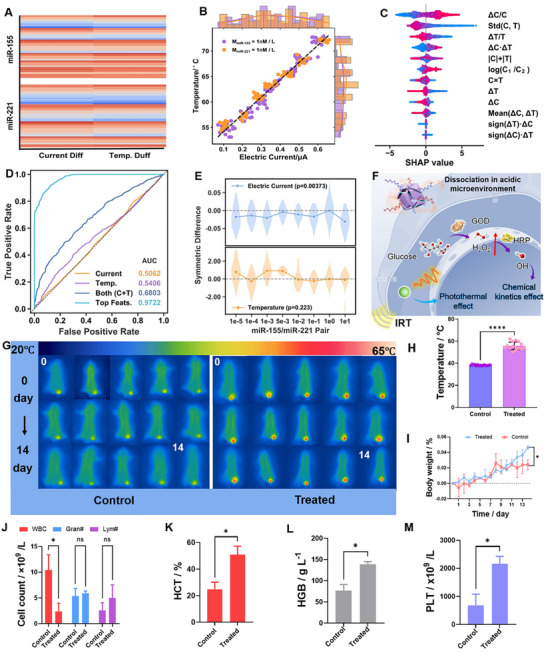
A) Data presentation of the dual‐modal detection for miRNA‐155 and miRNA‐221, B) Curves of current and temperature variations, C) SHAP (SHapley Additive exPlanations) value plot, D) Receiver operating characteristic (ROC) curve, E) Plots of current, temperature, and symmetrical difference analysis, F) Schematic illustration of the dissociation process of Ni‐ZIF‐8@GOD&HRP in the acidic tumor microenvironment and its mechanism of action on tumor cells, G,H) Infrared thermal imaging and temperature change curves of mice in the experimental and control groups (*P* < 0.0001), I) Body weight changes of mice during the treatment period, J–M) Bar charts of hematological parameters, including white blood cell count (WBC), granulocyte count (Gran#), lymphocyte count (Lym#), hemoglobin level (HGB), and platelet count (PLT), (*P < 0.05, **P < 0.01, ***P < 0.001, ****P < 0.0001, A value of *P* < 0.05 was considered statistically significant).

To enhance the accuracy and robustness of the self‐powered biosensing platform in distinguishing different miRNA expression states, this study introduces a deep learning approach for differential modeling and feature analysis of dual‐modal signals. The combinations of miRNA‐155 and miRNA‐221 at various expression levels were divided into multiple subgroups, and the Cartesian difference set was constructed based on the calculated current and temperature differences, forming the raw feature matrix. This strategy facilitates the simulation of scenarios involving changes in the expression of a single miRNA, thereby constructing an input feature set for supervised learning (**Figure** **6**A). Under symmetric conditions where both miR‐155 and miR‐221 were present at 1 nmol L^−1^ a good linear correlation between current and temperature was observed. The scatter plot and the marginal density distribution further demonstrate that the signal responses of the two are well matched under symmetric conditions (Figure 6B), reflecting the stability of the sensing system under equal concentration conditions. As shown in Figure 6C, feature importance analysis using an XGBoost classifier in combination with SHAP (SHapley Additive exPlanations) revealed that engineered features such as ΔC·ΔT, sign (ΔT)·ΔC, and log(C_1_/C_2_) achieved higher importance scores than the original features. These results indicate that interaction features played a crucial role in distinguishing changes in miRNA expression and outperformed the raw signals in classification tasks. Figure 6D presents the classification performance evaluated via ROC curves for different input feature sets. Figure S18 (Supporting Information) presents the confusion matrix, which is used to evaluate the classification performance of the model. When current and temperature signals were used independently, the AUC values were relatively low (0.5062 and 0.5406, respectively). However, combining both signals improved the AUC to 0.6803. Notably, the model achieved an AUC of 0.9722 when using the top‐ranked engineered features, demonstrating excellent predictive power and classification performance. To investigate whether swapping the concentrations of miRNA‐155 and miRNA‐221 (e.g., from (A, B) to (B, A)) leads to significant signal differences, we performed symmetric construction of signal differences and conducted a t‐test using zero as the baseline (Figure 6E). The results showed that the current difference significantly deviated from zero after the concentration swap (*p* = 0.00373), while the temperature difference did not reach statistical significance (*p* = 0.223). These findings suggest that the current signal is more sensitive to the order of concentration, whereas the temperature signal exhibits greater stability.

To verify the potential synergistic effect of CDT and PTT, preliminary in vitro experiments were conducted at different concentration levels, as shown in Figure S19 (Supporting Information). Cell viability was compared among groups treated with CDT alone, PTT alone, and the combined treatment (CDT + PTT). Bliss Δ values were calculated at each concentration point, and the results indicated that the combined treatment consistently exceeded the theoretical prediction (Δ > 0), demonstrating a synergistic effect. To systematically evaluate the synergistic antitumor efficacy of PTT and CDT, this study compared treated and control mice in terms of local temperature elevation, body weight dynamics, and multiple hematological and physiological parameters, thereby validating the comprehensive therapeutic performance of the proposed platform. As illustrated in Figure 6F, the Ni‐ZIF‐8@GOD&HRP nanoplatform gradually degrades under the acidic tumor microenvironment, releasing GOD to catalyze the conversion of glucose into H_2_O_2_. The generated H_2_O_2_ subsequently participates in a Fenton‐like reaction catalyzed by HRP to produce cytotoxic ·OH. This reactive oxygen species generation, in combination with photothermal ablation, enables a dual‐modality PTT/CDT strategy for effective tumor cell eradication. Infrared thermal imaging (Figure 6G,H; Figures S20 and S21, Supporting Information) demonstrated that the tumor temperature in the treated group rapidly increased to ≈60 °C upon laser irradiation, significantly higher than that in the control group, confirming excellent photothermal conversion efficiency and tumor‐targeting capability. As shown in Figure 6I, body weight monitoring revealed a significant decline in the control group after day 11, likely due to tumor‐induced systemic and tissue wasting.^[^ ^28^ ^]^ In contrast, the treated group exhibited a gradual increase in body weight, indicating an alleviation of cancer‐associated cachexia and suggesting the good biocompatibility of Ni‐ZIF‐8@GOD@HRP. Hematological analysis (Figure 6J–M) further supported the systemic therapeutic benefits. The elevated white blood cell count (1.25 × 10^10^ cells L^−1^) in the control group, indicative of cancer‐related inflammation (CRI), returned to normal levels (1.23 × 10^9^ cells L^−1^) in the treated group, accompanied by an increased lymphocyte ratio, suggesting attenuated inflammation and partial immune restoration. Compared to the control group, in which platelet counts were markedly reduced (4.05 × 10^11^ cells L^−1^), the treated group exhibited a significant increase to 2.35 × 10^12^ cells L^−1^, indicating reduced platelet consumption and enhanced bone marrow activity. Moreover, hemoglobin (HGB) levels increased to 143 g L^−1^ in the treated mice, compared to 87 g L^−1^ in the control group, with normalization of red blood cell indices, indicating alleviation of cancer‐induced anemia and recovery of hematopoietic activity. The biocompatibility of Ni‐ZIF‐8@GOD&HRP was further evaluated via hemolysis assays (Figure S22, Supporting Information). The results showed that the hemolysis rate remained below 5% across the concentration range of 0.5–4 mg mL^−1^, with no significant erythrocyte lysis observed, confirming the good blood compatibility of the nanoplatform. Meanwhile, ICP‐MS results demonstrated that the Ni content in the nanoparticles was low, with release levels far below the toxic threshold, confirming their good biosafetyb (Table S3, Supporting Information). In summary, this multifunctional therapeutic system integrates photothermal and chemodynamic modalities to achieve precise tumor ablation while simultaneously mitigating systemic inflammation, immunosuppression, and metabolic dysfunction, thus demonstrating strong potential for future multimodal cancer therapy applications.

## 3 Conclusion

In summary, this study successfully developed an intelligent cancer diagnosis and treatment platform featuring dual‐targeting capabilities, integrating passive/active targeting mechanisms, glucose‐dependent CDT, and near‐infrared PTT to achieve efficient and precise cancer diagnosis and therapy. The system exploits the unique metabolic microenvironment of tumor cells, wherein Ni‐doped MOF nanostructures catalyze glucose to generate H_2_O_2_, thereby triggering sustained ·OH release and significantly enhancing CDT efficacy. Meanwhile, the excellent photothermal properties of Ni‐MOF enable localized hyperthermia under infrared laser irradiation, synergistically promoting tumor cell inactivation. Both in vitro and in vivo experiments confirmed the platform's outstanding therapeutic efficiency and biocompatibility. DFT simulations further elucidated the electronic structure characteristics and photothermal conversion mechanism of the Ni‐MOF material, providing theoretical support for its catalytic activity. Additionally, machine learning‐assisted modeling was employed to effectively evaluate miRNA expression changes and therapeutic responses, offering data support for personalized precision medicine. The constructed dual‐modal intelligent responsive diagnostic and therapeutic platform offers a novel approach for next‐generation precision cancer therapy, demonstrating promising translational prospects and broad applicability for future research.

## 4 Experimental Section

### Preparation of Ni‐ZIF‐8@GOD&HRP

First, 0.002 mol of Zn(NO_3_)_2_·6H_2_O and 0.003 mol of Ni(NO_3_)_2_·6H_2_O were dissolved in 40 mL of methanol and vigorously stirred for 15 min to obtain solution A. Separately, 0.04 mol of 2‐methylimidazole (2‐MIM) was dissolved in 50 mL of methanol and vigorously stirred for 15 min to obtain solution B. Next, 2 mL of GOD solution (2 mg mL^−1^) and 2 mL of HRP solution (2 mg mL^−1^) were dissolved in deionized water to prepare solution C. Then, 4 mL of solution C was mixed with 50 mL of solution B to obtain solution D. Finally, solution A was dropwise added into solution D at room temperature under vigorous stirring for 12 h. The resulting precipitate was washed three times with methanol.

### Bio‐Conjugate Preparation

First, 60 µL of CP‐DNA‐155 (1 nmol L^−1^), 3 µL DEPC, and 5 µL EDC/NHS (1 mg mL^−1^) were mixed at room temperature for 1 h. Meanwhile, 400 µL of GOD (5 mg mL^−1^) was combined with 1 mg of sulfo‐SMCC. The CP‐DNA‐155‐GOD complex was then prepared by oscillating the mixture for 1 h and storing it at 4 °C. The same procedure was applied to prepare the CP‐DNA‐221‐GOD complex. Next, 50 µL of the Ni‐ZIF‐8/AuNPs complex was dropped onto the CP electrode and dried at 37 °C The dried electrodes were immersed in the EDC/NHS mixture (1 mg mL^−1^) for 0.5 h. After washing with deionized water, 30 µL of DNA‐155 and DNA‐221 (1 nmol L^−1^) were added to the electrode surface and reacted at 4 °C for 12 h. This process yielded the DNA‐221/DNA‐155/Ni‐ZIF‐8/AuNPs/CP bioanodes. A 50 µL Ni‐ZIF‐8/AuNP drops were added to the CP electrode, dried at 37 °C, and incubated with 30 µL EDC/NHS (1 mmol L^−1^) to block non‐specific adsorption. Wash with deionized water to remove excess EDC/NHS. The electrode was then incubated with 30 µL of BOD at 4 °C overnight to obtain a BOD/ Ni‐ZIF‐8/AuNPs/CP bio‐cathode.

### Sensor Preparation and Test Conditions

The base sequence used in the experiment is shown in Table S1 (Supporting Information). The PBS solution containing 5 mmol L^−1^ glucose was selected as the electrolyte solution, and the bio‐anode and bio‐cathode were used to construct the self‐powered biosensor. In the bio‐anode, DNA‐155 performs complementary base pairing with part of the target miRNA‐155, and CP‐DNA‐155‐GOD performs complementary base pairing with another part. DNA‐221 performs complementary base pairing with one part of the target miRNA‐221, and CP‐DNA‐221‐GOD performs complementary base pairing with another part. GOD was fixed on the surface of the biological anode for catalytic reaction. GOD catalyzes glucose oxidation, releases electrons to the bio‐cathode through the external circuit for O_2_ reduction reaction, and generates an instantaneous current signal response.

### In Vivo Experiments

Six‐week‐old male C57BL/6 mice were used to establish a subcutaneous prostate cancer xenograft model. In the treatment group, NIR irradiation ( 2W cm^−2^) was applied to the tumor site at a distance of 1 cm for 30 min after each administration, with temperature changes monitored using an infrared thermal imaging camera. The treatment was conducted once daily at a fixed time for 14 consecutive days, during which body weight was recorded regularly. Mice in the control group received PBS injections, while all other experimental procedures remained consistent. At the end of the study, mice were anesthetized, and blood samples were collected. Serum was isolated and subjected to biochemical analysis using an automated biochemical analyzer.

## Conflict of Interest

The authors declare no conflict of interest.

## Supporting information



Supporting Information
