# Supplementary material for: A Dual‐Target Recognition System Based on Acid‐Degradable Ni‐MOF and Aptamer Guidance for Precise Tumor Diagnosis and Combined Therapy
Source: Adv Sci (Weinh). 2025 Sep 24;12(42):e12838. doi: 10.1002/advs.202512838 (PMC12622455; doi:10.1002/advs.202512838)
Supplement: Supplementary file 1 — Supporting Information [file ADVS-12-e12838-s001.docx]

**Supplementary Information**

**A Dual-Target Recognition System Based on Acid-Degradable Ni-MOF and Aptamer Guidance for Precise Tumor Diagnosis and Combined Therapy***Jing Xu^1^, Hanxiao Chen^1^, Yifang Tao^1^, Hong Wang^1^, Zhenlong Wang^1^, Yuquan Xue^1^, Delai Fu^1^, Huan Pang^2*^, Li Xue^1*^*
1 Department of Urology, The Second Affiliated Hospital of Xi'an Jiaotong University, Xi’an 710004, China
2 School of Chemistry and Chemical Engineering, Yangzhou University, Yangzhou 225009, China

*Corresponding author to one of the following:
Huan Pang, Yangzhou University, Email: huanpangchem@hotmail.com
Li Xue, The Second Affiliated Hospital of Xi'an Jiaotong University, Email: xueli1979@xjtu.edu.cn

**Chemicals**

The DNA sequences of oligonucleotides (**Table** S1) were purchased from Shanghai Shenggong Biotechnology Co., Ltd. 1-ethyl-3-(3’-dimethylaminopropyl) carbodiimide (EDC, ≥ 98%), N hydroxysuccinimide (NHS, ≥ 98%), glucose oxidase (GOD, BR, 100-250 U/mg), diethylprocarbonate (DEPC, ≥ 99%), chloroauric acid tetrahydrate (HAuCl_4_·3H_2_O, ≥ 99%), 4-(N maleimidomethyl) cyclohexane-1-carboxylic acid 3-sulfo-N-hydroxysuccinimide ester sodium salt (sulfo-SMCC, ≥ 99%), phosphate buffered saline (PBS), sodium citrate (≥ 99%), monosodium dihydrogen phosphate (NaH_2_PO_4_, ≥ 99%), ethanol (C_2_H_6_OH, ≥ 99%), Zinc nitrate hexahydrate (Zn(NO_3_)_2_·6H_2_O) and disodium hydrogen phosphate (Na_2_HPO_4_, ≥ 99%) were obtained from Sinopharm Chemical Reagent Co. Ltd (Shanghai, China). Nickel nitrate (Ni(NO_3_)_2_·6H_2_O) was purchased from Shanghai Hengxin Chemical Reagent Co., Ltd. The carbon paper (CP) (WOS1009) was purchased from Sigma-Aldrich. Potassium ferrocyanide trihydrate (K_4_[Fe(CN)_6_], ≥ 99.95%), potassium ferricyanide (K_3_[Fe(CN)_6_], ≥ 99.5%), potassium chloride (KCl, ≥ 99. 5%), NaOH (≥ 97%), 6-Mercapto-1-hexanol (MCH, 97%), acetic acid (CH_3_CH_2_COOH, ≥ 99.7%), sodium acetate solution (CH_3_CH_2_COONa, 99%), 3,3',5,5'-Tetramethylbenzidine (TMB, 99.0%), 3,3'-Diaminobenzidine (DAB, 99%), Fluorescein isothiocyanate (FITC, 97%), sodium chloride (AR, 99.5%), tris buffer solution (0.01 M) were purchased from McLean Biochemical Reagent Co., Ltd. Horseradish peroxidase (HRP, ≥ 250 U/mg) was obtained from Aladdin Biochemical Technology Co., Ltd.

The [Fe(CN)_6_]^3-/4-^ solution for the electrochemical tests consisted of 10 mM K_4_Fe(CN)_6_, 10 mM K_3_Fe(CN)_6_, and 0.1 M KCl. The buffer solutions for the experiment were as follows: phosphate buffered solutions (PBS) consisted of 0.1 M NaCl, 0.1 M Na_2_HPO_4_, and 0.1 M NaH_2_PO_4_ (pH 7.4).

**Experimental Section**

**Preparation of Ni-ZIF-8^[1]^**

0.002 mol of Zn(NO_3_)_2_·6H_2_O and 0.003 mol of Ni(NO_3_) _2_·6H_2_O were dissolved in 40 mL of methanol and vigorously stirred for 15 min to obtain solution A. Separately, 0.04 mol of 2-methylimidazole (2-MIM) was dissolved in 50 mL of methanol and vigorously stirred for 15 min to obtain solution B. Then, at room temperature, solution A was dropwise added into solution B with vigorous stirring for 12 hours. Finally, the precipitate was washed three times with methanol and centrifuged, followed by drying at 60 °C.

**Antibacterial Assay**

The antibacterial experiment was divided into two groups: a control group (NIR irradiation only) and a Ni-ZIF-8@GOD&HRP+NIR group. Once a large number of bacterial colonies had grown on the agar plates, individual colonies were selected using an inoculation loop and diluted in normal saline. A 1 mL mixture of bacterial suspension and Ni-ZIF-8@GOD&HRP was prepared and then exposed to NIR laser irradiation (808 nm, 1 W/cm^2^, 25 min). The mixture was subsequently incubated for 4 hours. After incubation, DMAO/PI staining was performed, and confocal imaging was used to capture the antibacterial effects. Additionally, 100 μL of the bacterial suspension was evenly spread onto LB agar plates and incubated for 24 hours for colony counting.

**Synthesis of Au NPs**
Gold nanoparticles (Au NPs) were synthesized using the sodium citrate reduction method. Briefly, 100 mL of 10% HAuCl_4_·3H_2_O solution was diluted with 50 mL of ultrapure water and heated to boiling. Then, 100 μL of 1% sodium citrate solution was added to the boiling mixture under continuous stirring. The solution was stirred until it turned wine-red, and heating was maintained for an additional 10 minutes. After cooling to room temperature, the resulting Au NPs suspension was stored at 4 °C in the dark for further use.

**Fluorescent Labeling of GOD and HRP**

GOD (2 mg/mL) and HRP (2 mg/mL) were dissolved in PBS solution (10 mM, pH 7.4) containing FITC (2 mg/mL) and RhB (2 mg/mL), respectively, and stirred in the dark for 12 hours. Subsequently, the mixtures were dialyzed against deionized water for 48 hours using dialysis bags (1 kDa molecular weight cutoff). Finally, the labeled GOD and HRP were lyophilized and stored at –20 °C for future use.

**Determination of Enzyme Loading**

Rhodamine B-labeled horseradish peroxidase (RhB-HRP) and fluorescein isothiocyanate-labeled glucose oxidase (FITC-GOD) were employed as fluorescent probes to quantitatively determine the enzyme loading content (wt%) of in situ encapsulated horseradish peroxidase and glucose oxidase. A series of standard solutions of RhB-HRP and FITC-GOD with concentrations ranging from 5 to 30 μg mL^-1^ were prepared, and their fluorescence emission spectra were recorded over the range of 425-700 nm. The fluorescence intensities at the maximum emission wavelengths were used to construct calibration curves, both of which exhibited excellent linearity (R^2^ = 0.995). Ni-ZIF-8@GOD&HRP composites were synthesized via the in situ encapsulation method. After centrifugation, the supernatant and all sequential washing solutions were collected, and their fluorescence intensities were measured. The concentrations of RhB-HRP and FITC-GOD in each fraction were determined from the corresponding calibration curves. The enzyme loading content (wt%) was calculated according to the following equation:

Loading content = [(M _initial enzyme_−M _enzyme in supernatant and washings_)/M _materials_] × 100%

**Characterization**

The voltage range for cyclic voltammetry (CV) measurements was set from -0.8 V to 0 V, using 10 mM phosphate-buffered saline (PBS) as the electrolyte solution. For linear sweep voltammetry (LSV), the voltage range was also -0.8 V to 0 V, with 10 mM PBS solution as the electrolyte. Current measurements were performed using a UNI-T multimeter, with 10 mM PBS solution (pH = 7.40) as the electrolyte. Electrochemical impedance spectroscopy (EIS) was conducted over a frequency range of 100 mHz to 100 kHz, using 100 mM PBS solution (pH = 7.40) containing 5.0 mM [Fe(CN)_6_]^3-^/^4-^ and 0.1 mM KCl as the electrolyte.

**Cell Thawing Procedure**

Remove the cryovial from dry ice and immediately place it in a 37 °C water bath, gently agitating it intermittently to accelerate thawing. Once thawed, remove the cryovial from the water bath, disinfect the exterior with 75% ethanol, and dry it. Open the cap and gently transfer the cell suspension to a centrifuge tube. Add at least 5 times the volume of pre-warmed complete culture medium and mix thoroughly. Centrifuge at 1000 rpm for 5 min. Discard the supernatant and resuspend the cell pellet in 5-6 mL of complete medium. Perform cell counting, adjust the cell density, and seed into a T25 culture flask. Incubate the flask in a 37 °C incubator under static conditions. Replace the complete medium the next day and continue culturing.

**Cell Passage**

When cell confluence reaches approximately 90% in the incubator, passaging can be performed. For a T25 culture flask, aspirate the complete medium and gently rinse the cells once with PBS buffer. Add 0.5 mL of 0.25% trypsin containing EDTA and incubate for 2 minutes (the exact digestion time may vary depending on the cell type). Then, add 1 mL of complete medium to stop the digestion and detach the cells. Centrifuge at 1000 rpm for 5 minutes. Discard the supernatant, collect the cell pellet, and evenly seed into two T25 flasks, each with 5 mL of complete medium.

**Note:** Avoid over-digestion. Ensure that all serum components are thoroughly removed before digestion. Always use fresh complete medium to terminate trypsin digestion; the original culture medium should not be reused for digestion termination or subculturing.

**Cell Counting Analysis**

After digestion, fully resuspend and disperse the cells into a single-cell suspension. Transfer 1 mL of the suspension into a clean EP tube. Gently wipe the surface of the hemocytometer and the coverslip with an alcohol swab. Moisten the edges of the coverslip slightly and carefully place it over the central area of the hemocytometer so that the covers the loading chambers evenly. Resuspend the cell suspension in the EP tube by pipetting up and down again to ensure even distribution. Pipette 20 μL of the cell suspension and carefully load it at the edge of the coverslip (upper or lower edge). The liquid will be drawn into the chamber by capillary action. Adjust the microscope and observe under a 10× objective lens. Count the number of viable cells (those not stained by trypan blue) according to standard cell counting procedures. After obtaining the total number of cells in four large squares, calculate the cell concentration using the following formula:

Cell concentration (cells/mL) = (Total cell number in four large squares4) × 10,000. To calculate the total number of cells, multiply the volume of the resuspended culture medium by the calculated cell concentration:

Total cell number = Volume of resuspended medium (mL)×Cell concentration (cells/mL)

**Establishment of Subcutaneous Tumor Model**

Male C57BL/6 mice (6 weeks old) were purchased from the Hubei Provincial Laboratory Animal Research Center and housed under a controlled 12-hour light/12-hour dark cycle with ad libitum access to food and water. The fur on the right side of each mouse was shaved, and the skin was disinfected with an alcohol swab prior to injection. A total of 5×10^5^RM1 cancer cells were then subcutaneously injected into the anterior region of the right hind limb of each mouse. Five mice were used per group. When the average tumor volume reached 100 mm³, the mice were randomly divided into two groups for experimental procedures.

**Animal Ethics Statement**

All mice were purchased from the Hubei Provincial Laboratory Animal Research Center. All animal procedures were approved by the Laboratory Animal Center of Xi’an Jiaotong University and were conducted under the authorization for biomedical research involving animals (XJTUAE2025.1016).

**Density Functional Theory (DFT) Computational Methods and Parameters for Geometry Optimization**

The first-principle based geometry optimization calculations were carried out within density-functional theory (DFT), implemented in the Vienna Ab Initio Simulation Package (VASP) code, using the frozen-core projector augmented-wave (PAW) method to describe the interaction between the atomic cores and the valence electron density. The exchange-correlation potential was approximated within the generalized gradient approximation (GGA) using the Perdew-Burke-Ernzerhof (PBE) functional which was used to calculate electron-electron exchange and correlation interactions. A kinetic energy cutoff of 450 eV was chosen to ensure complete convergence. The Brillouin zone was sampled with the k-grid of 1×1×1. The conjugate gradient (CG) method was used for structural relaxation, simultaneously minimizing total energy and interatomic forces. The convergence tolerance for the total energy was set at 10^-5^ eV, and the residual force on each atom was limited to less than 0.05 eV/Å. Additionally, dispersion-corrected DFT-D3 schemes were employed to account for van der Waals interactions. The ZIF-8 model was obtained from the CCDC database.

**Statistical Analysis**
Certain datasets were analyzed using GraphPad Prism 9.0 software, with results expressed as mean ± standard deviation (SD). Statistical analyses were performed using one-way or two-way ANOVA and unpaired two-tailed Student’s *t*-tests as appropriate. Significance was defined as **P* < 0.05, ***P* < 0.01, ****P* < 0.001, *****P* < 0.0001. A value of *P* < 0.05 was considered statistically significant.

**
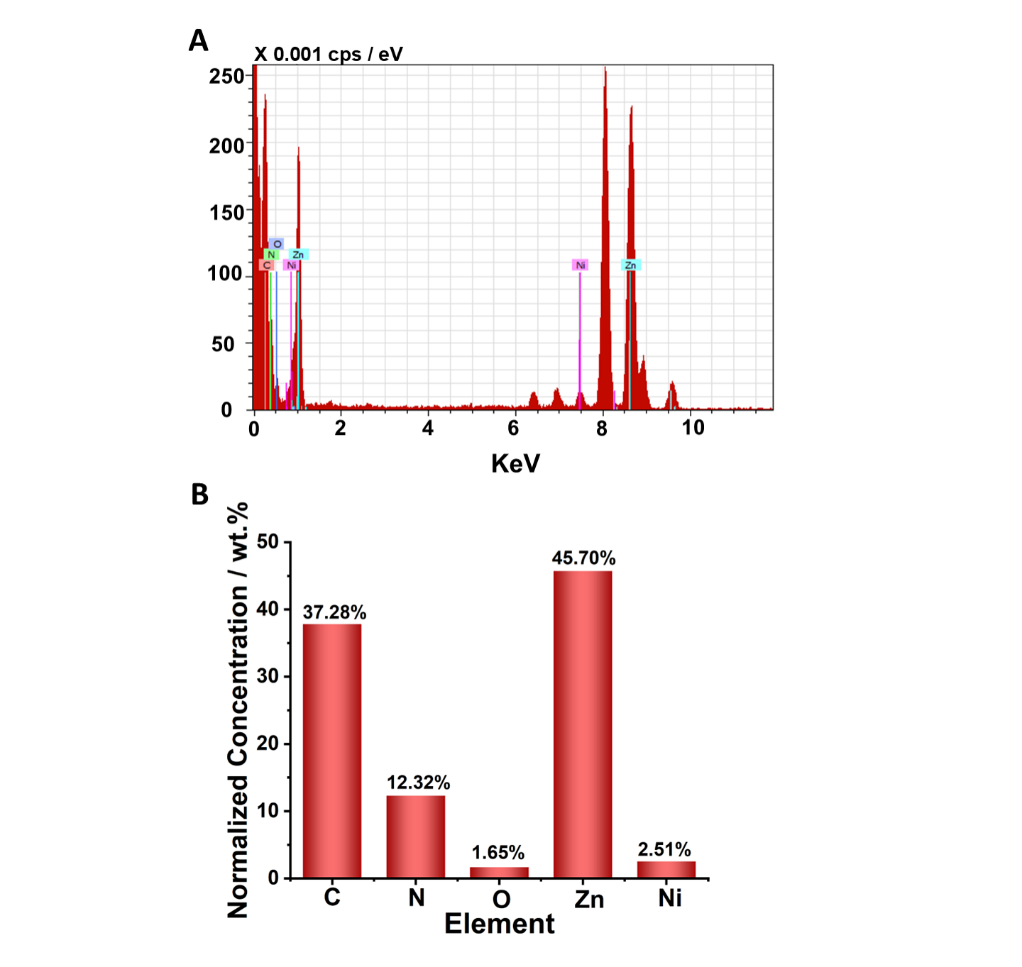
Figure** S1. (A, B) TEM-EDS elemental analysis of the sample.


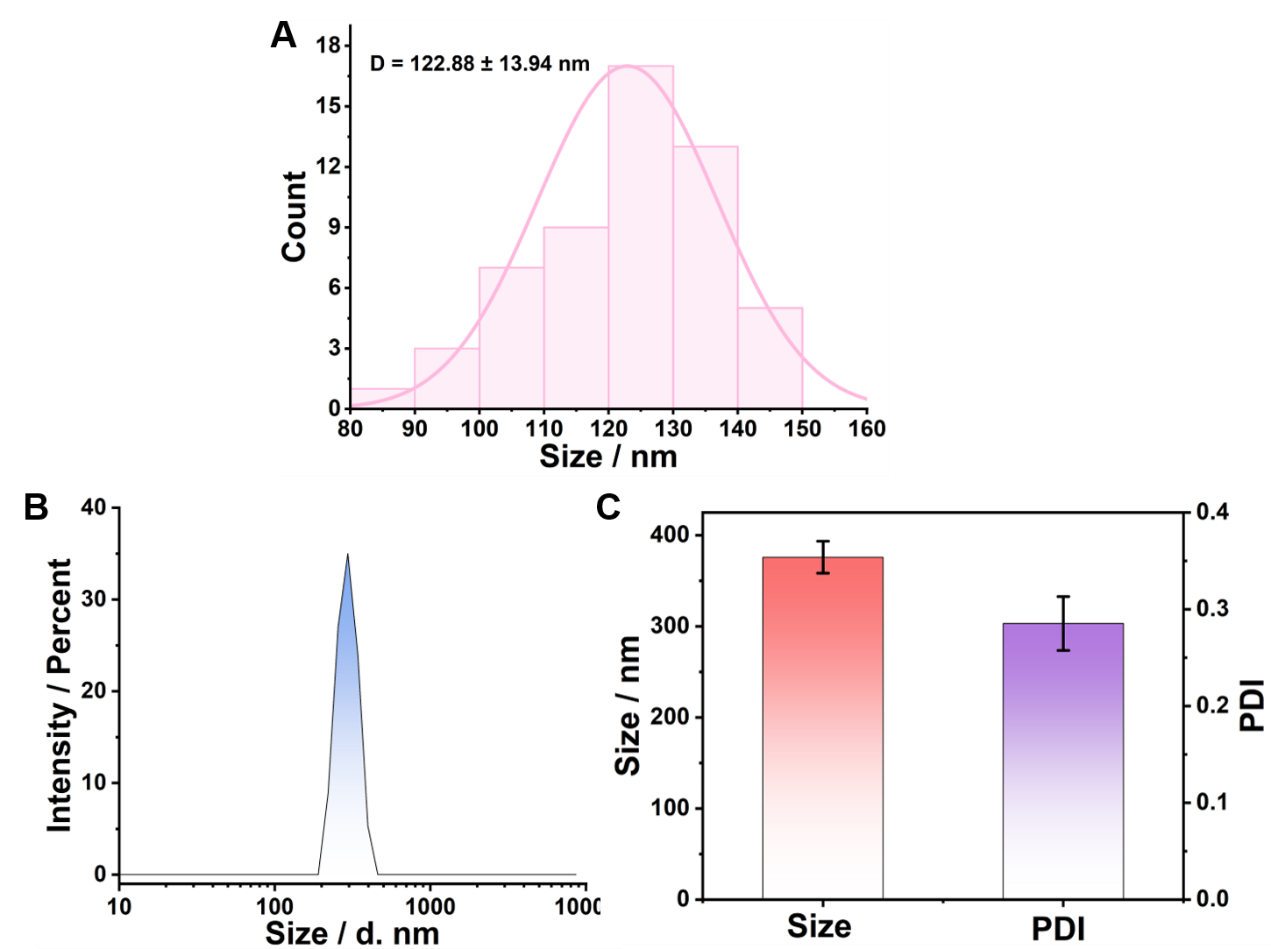


**Figure** S2. (A) Dynamic light scattering (DLS) intensity distribution profile of the nanoparticles dispersed in aqueous medium, (B) Average hydrodynamic diameter and polydispersity index (PDI) obtained from DLS measurements. Error bars represent standard deviations from three independent measurements.

**
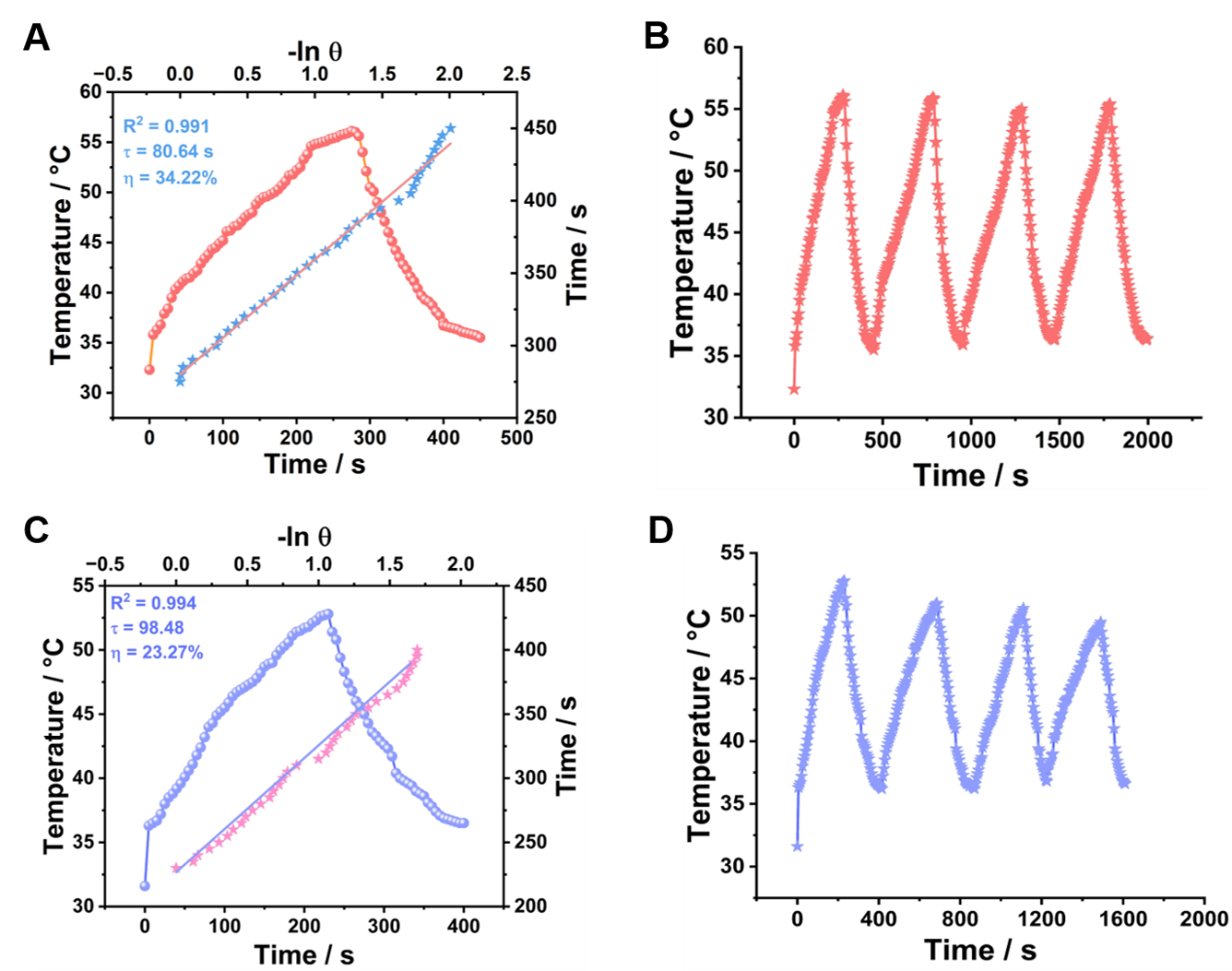
**

**Figure** S3. (A) Photothermal heating and cooling curve of Ni-ZIF-8@GOD&HRP solution at pH 7.4 under NIR laser irradiation, (B) Photothermal stability of Ni-ZIF-8@GOD&HRP at pH 7.4 over four cycles of laser on/off irradiation, indicating excellent photothermal durability, (C) Photothermal heating and cooling curve of Ni-ZIF-8@GOD&HRP solution at pH 5.0 under NIR laser irradiation, (D) Photothermal stability of Ni-ZIF-8@GOD&HRP at pH 5.0 over four cycles of laser on/off irradiation, showing decreased stability compared to pH 7.4.

**
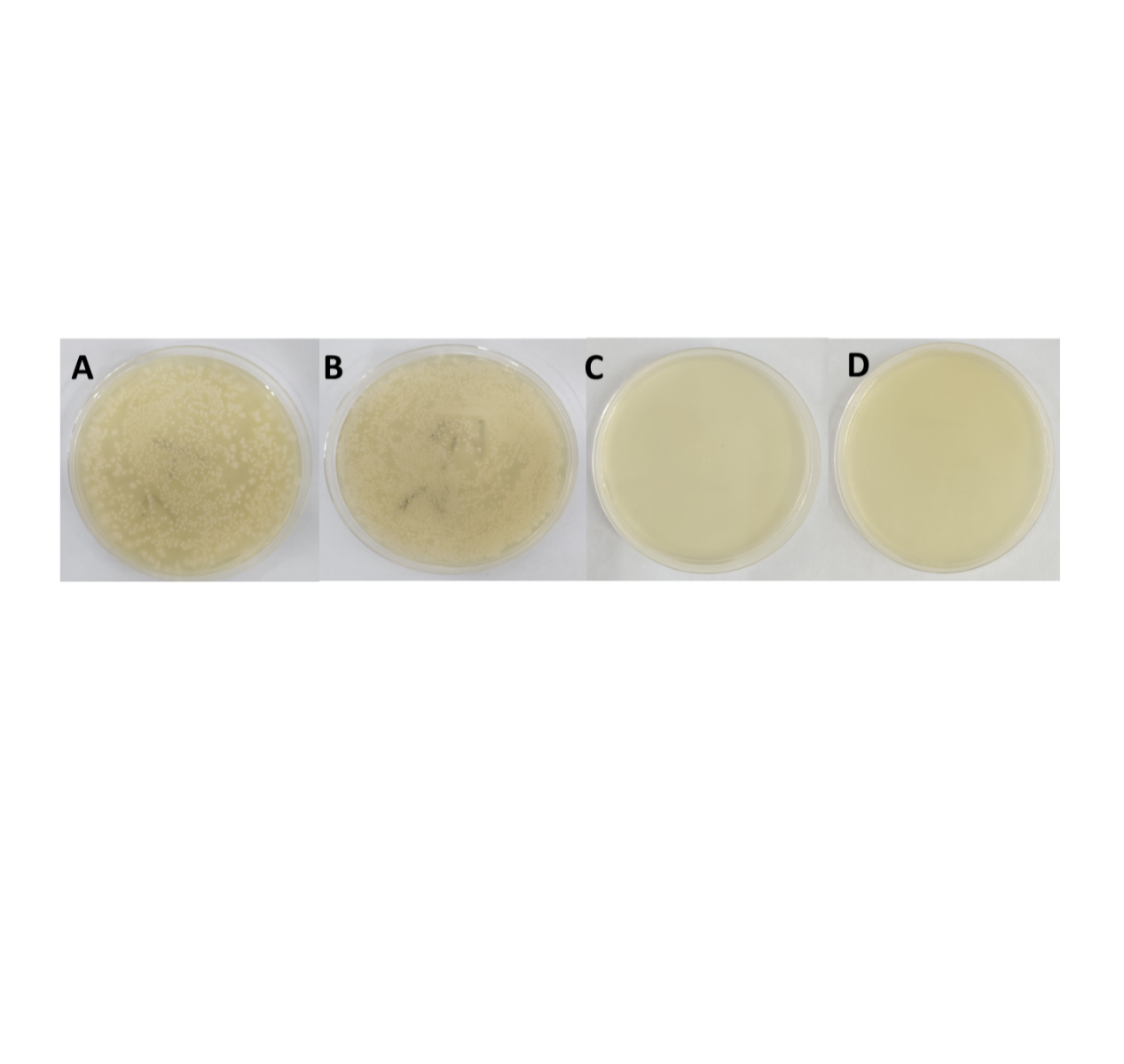
Figure** S4. Antibacterial Assay Under Near-Infrared Irradiation: Agar plate images of Salmonella typhimurium under different treatment conditions (with or without Ni-ZIF-8@GOD&HRP) following near-infrared (NIR) laser exposure.


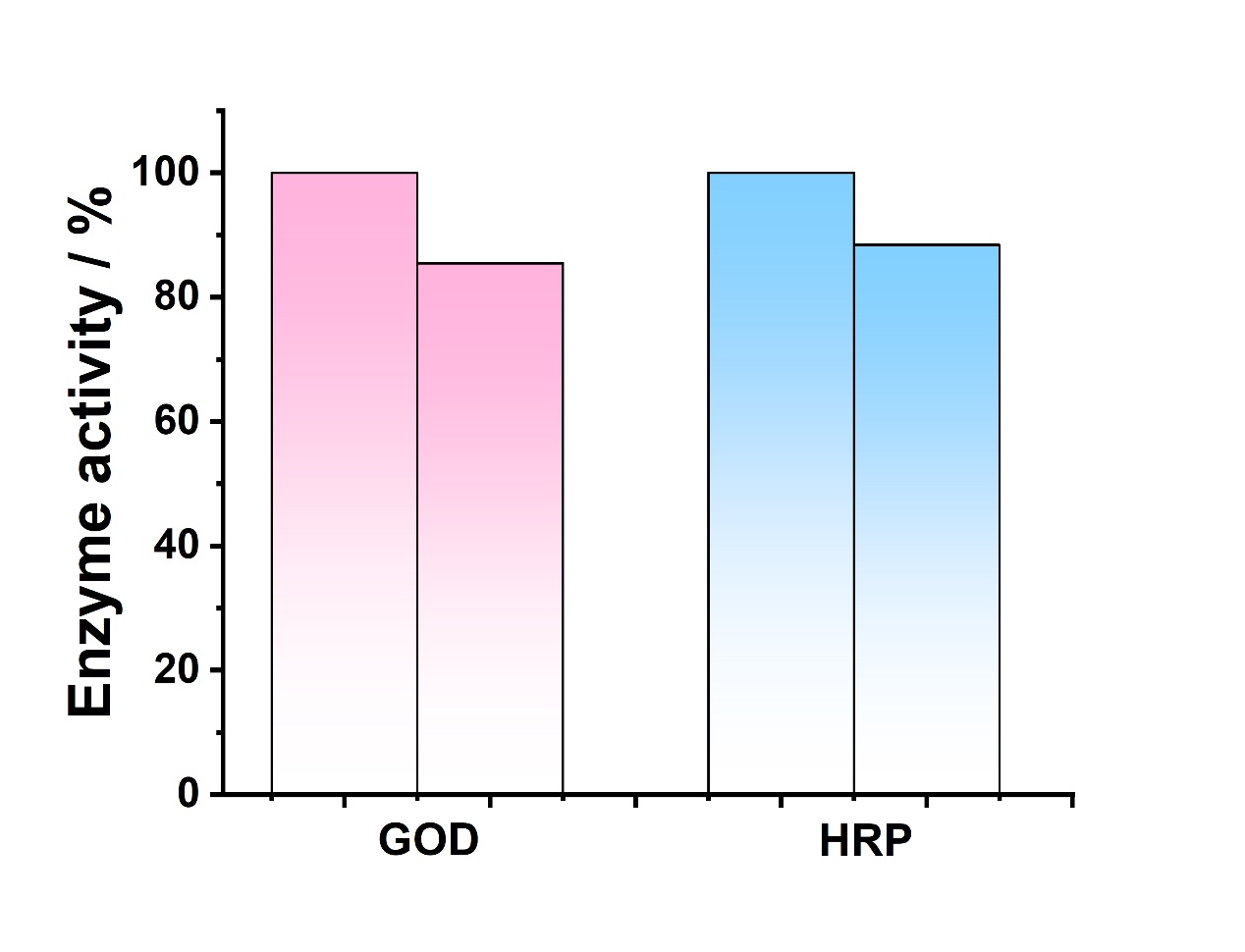


**Figure** S5. GOD and HRP enzyme activities remain well preserved after treatment at 50 °C for 10 min.


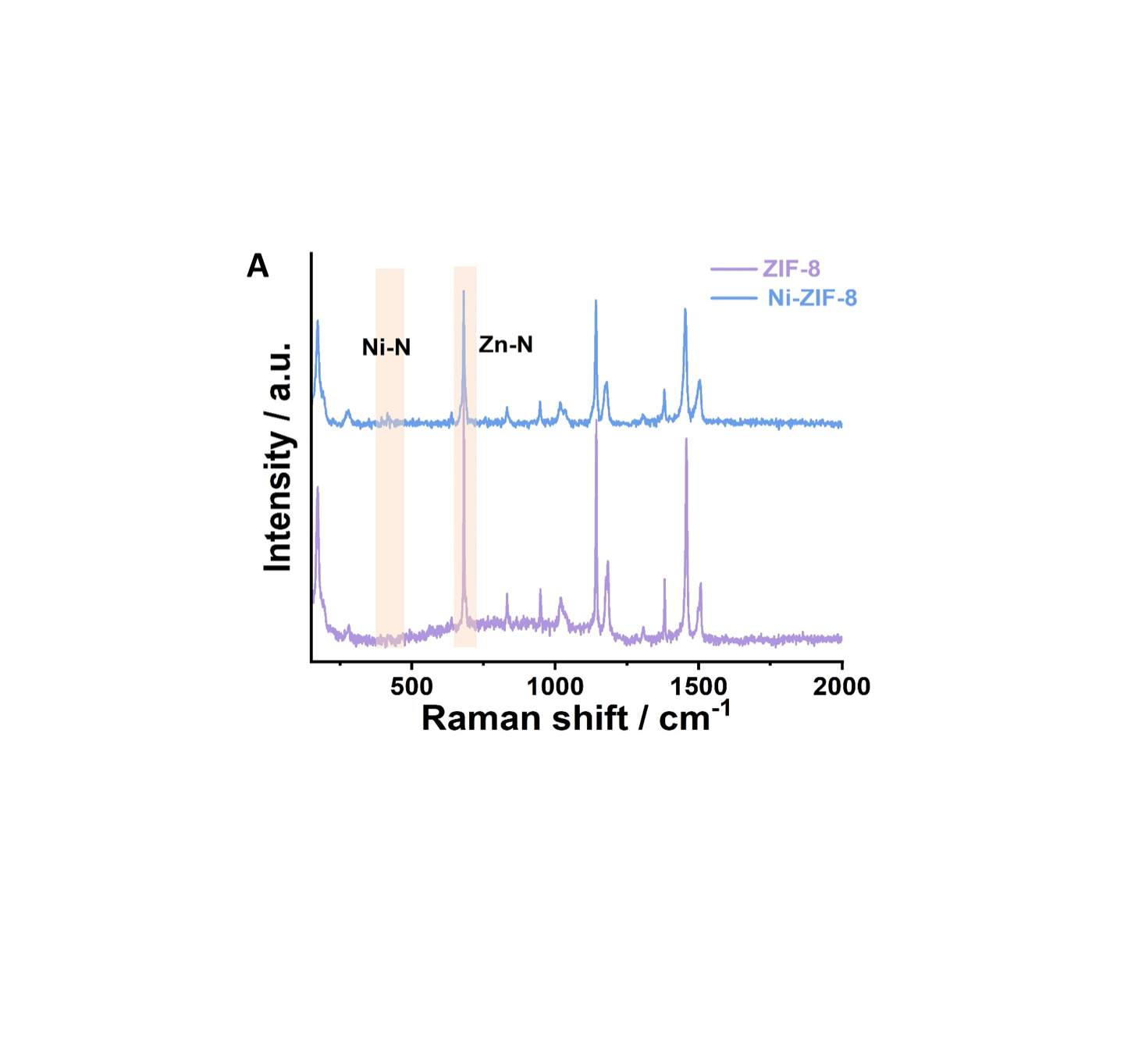
Figure S6. Raman Spectrum of ZIF-8 and Ni-ZIF-8.


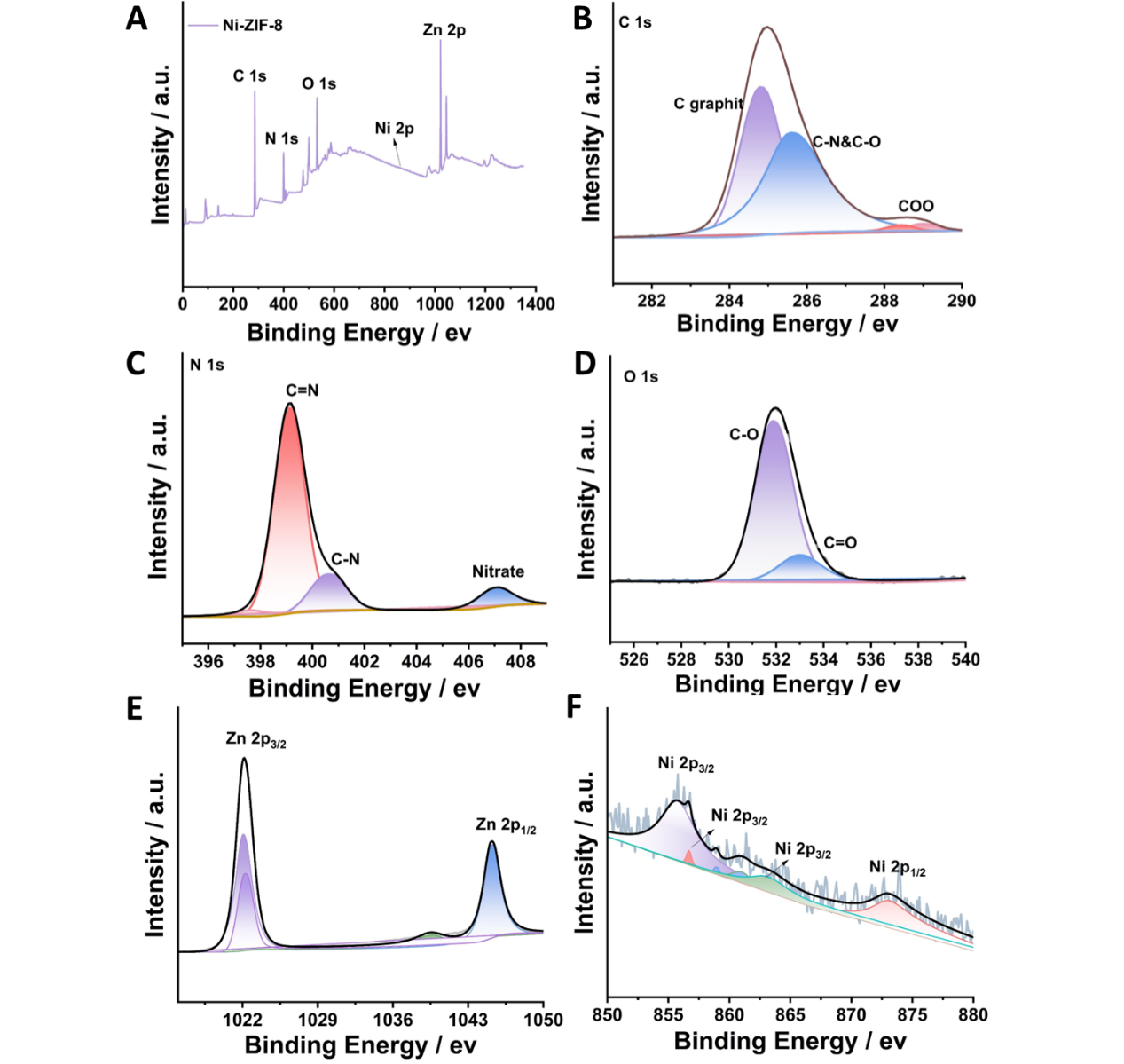
Figure S7. X-ray Photoelectron Spectroscopy (XPS) survey spectrum and high-resolution spectra of Ni-ZIF-8, including C 1s, N 1s, O 1s, Zn 2p, and Ni 2p.

**
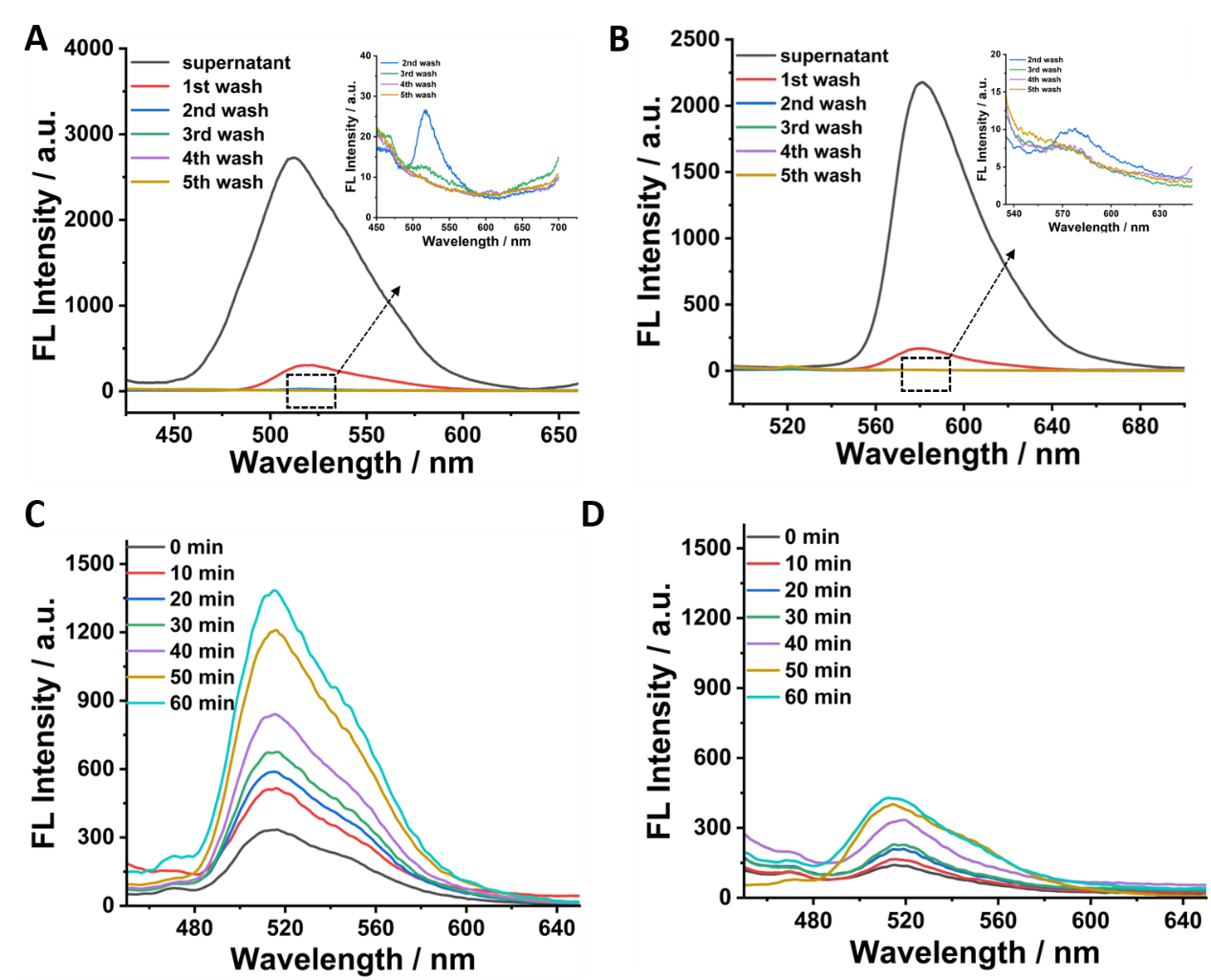
**

**Figure** S8. Fluorescence intensity of the supernatant and sequential washing solutions (1st-5th wash) obtained during the determination of loading content for (A) horseradish peroxidase and (B) glucose oxidase. The supernatant was collected immediately after the initial enzyme loading, and the washing solutions were obtained sequentially to remove unbound enzymes, (C, D) Time-dependent release profiles of the fluorescently labeled dual enzymes from Ni-ZIF-8@GOD&HRP under pH 5.0 and 7.4 conditions.


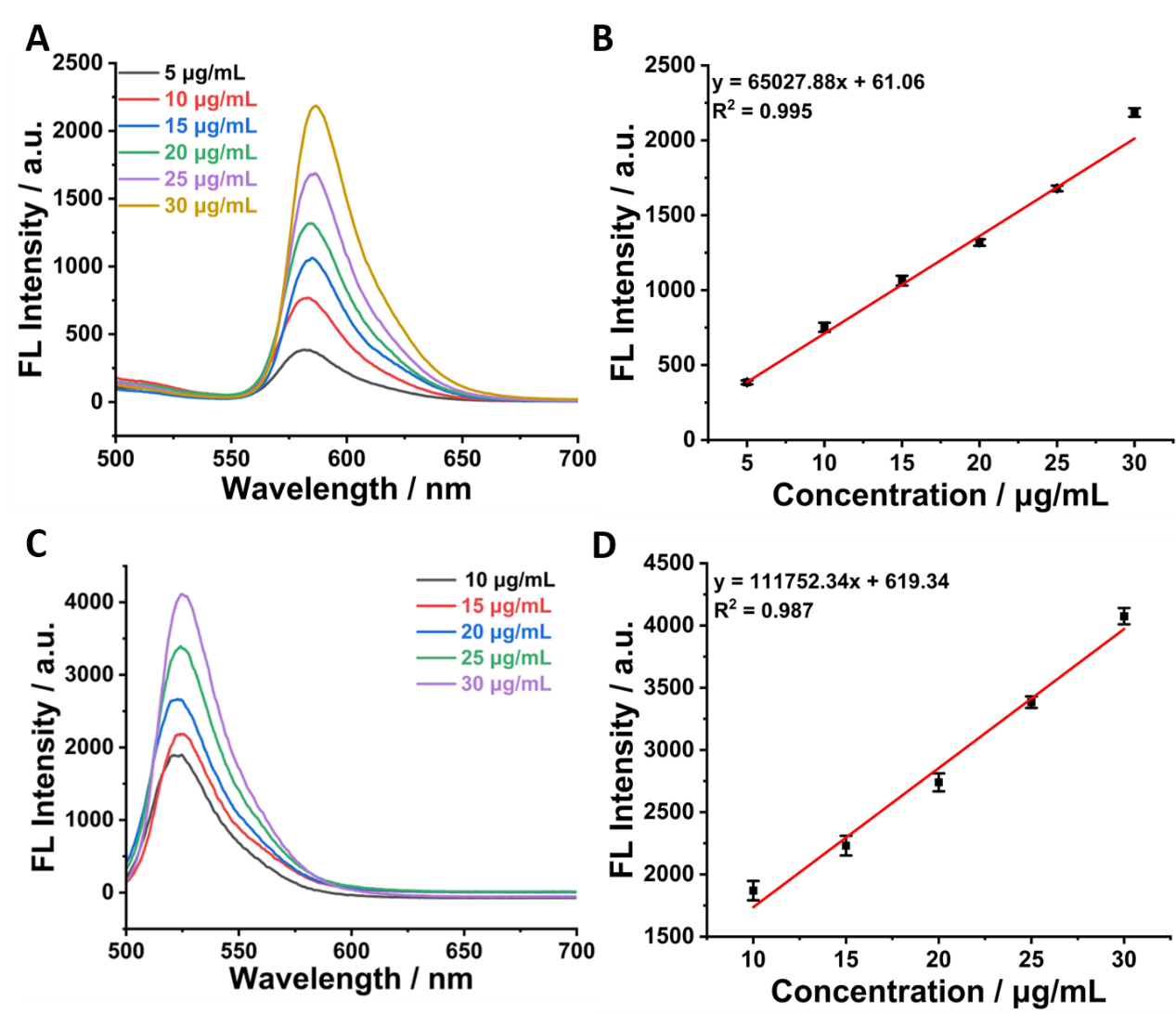


**Figure** S9. Fluorescence standard curves of RhB-labeled horseradish peroxidase (RhB-HRP) and FITC-labeled glucose oxidase (FITC-GOD) for determining enzyme loading content. (A) Fluorescence emission spectra of RhB-HRP at various concentrations (5-30 μg/mL) and (B) the corresponding linear calibration curve (R^2^ = 0.995). (C) Fluorescence emission spectra of FITC-GOD at various concentrations (10-30 μg/mL) and (D) the corresponding linear calibration curve (R^2^ = 0.987).


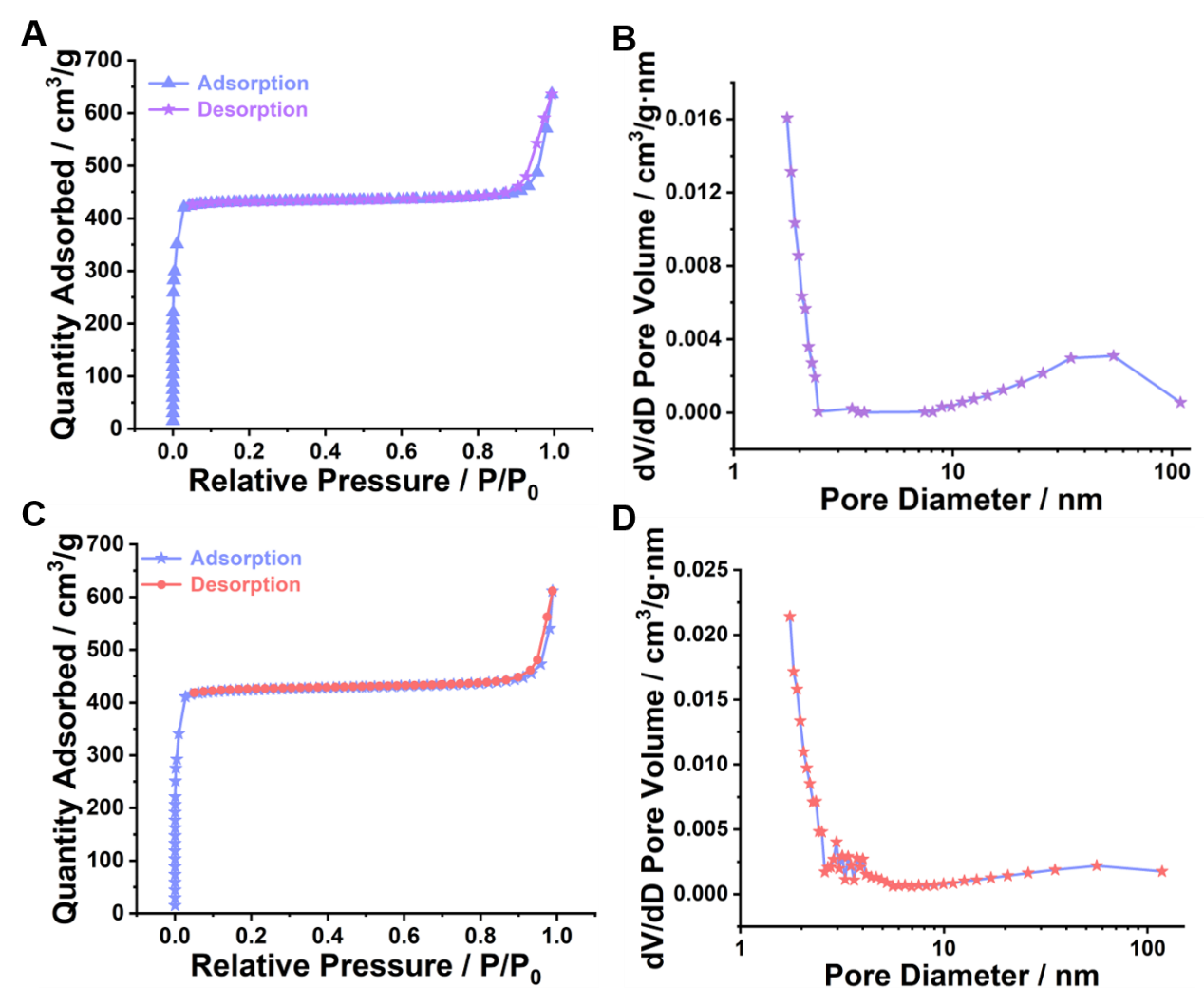


**Figure** S10. Nitrogen adsorption-desorption isotherms of Ni-ZIF-8 before enzyme (GOD and HRP) encapsulation (A) and the corresponding pore size distribution (B), and nitrogen adsorption-desorption isotherms of Ni-ZIF-8 after enzyme encapsulation (C) with the corresponding pore size distribution (D). The slight decrease in specific surface area and average pore size after enzyme loading indicates successful encapsulation while preserving the overall structure.


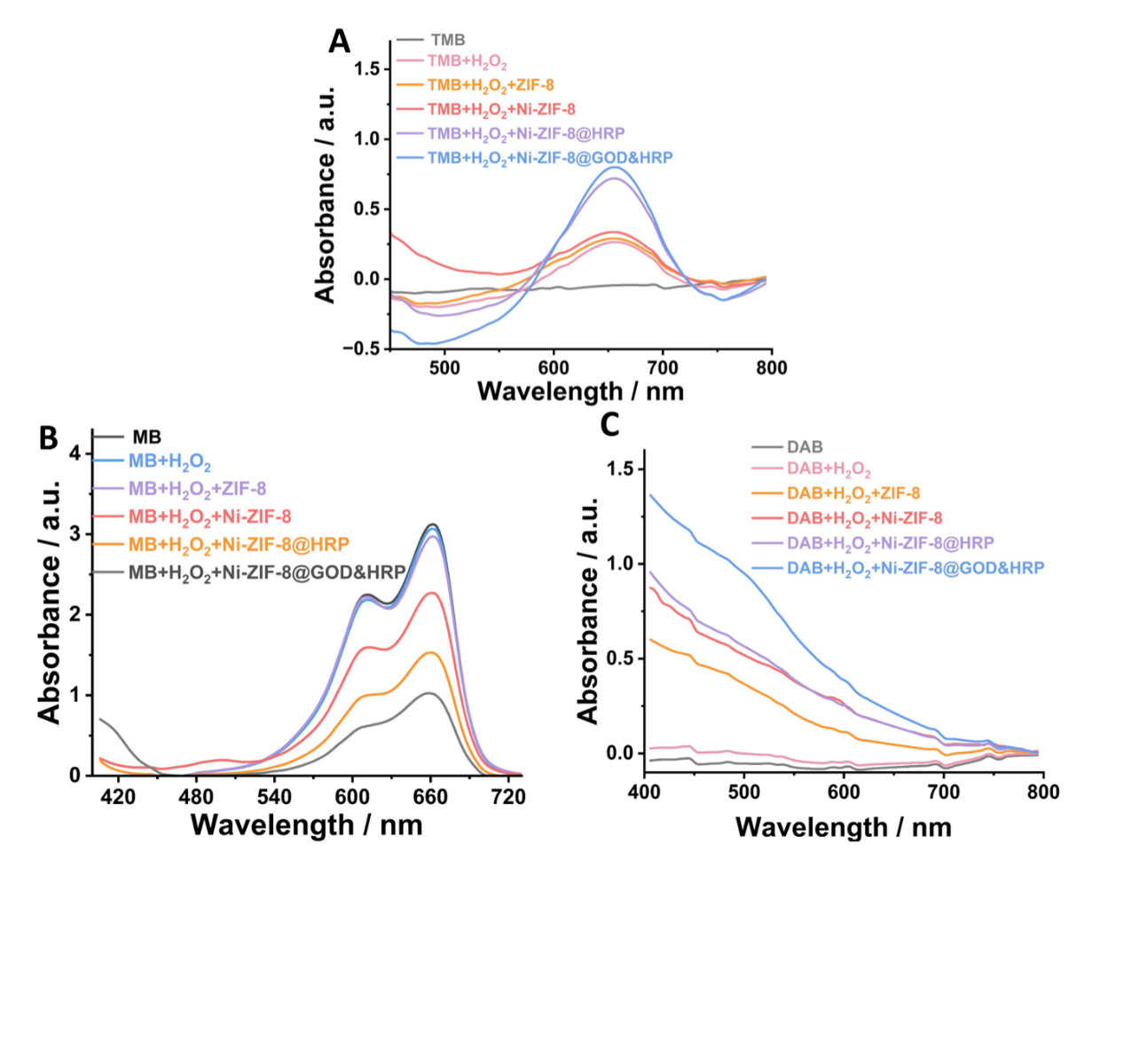


**Figure** S11. UV-Vis spectra of different reaction systems using TMB, MB, and DAB as chromogenic substrates.

**
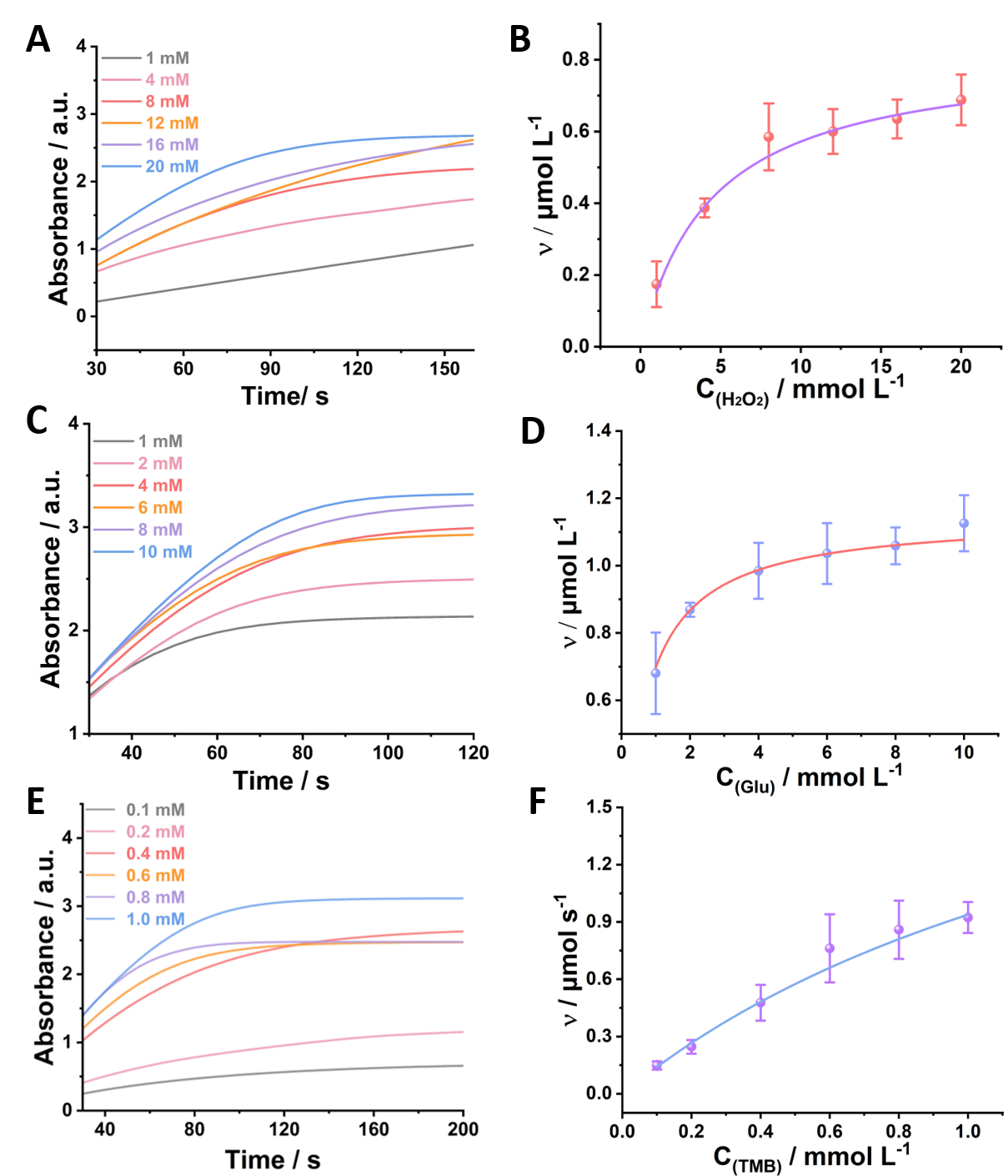
**

**Figure** S12. Evaluation of the Catalytic Activity of the Nanocomposite: (A) Time-dependent curves for different concentrations of hydrogen peroxide (H_2_O_2_) solutions, (B) Michaelis-Menten kinetics of the catalytic reaction with 10 mM glucose and 0.6 mM TMB under varying H₂O₂ concentrations in the presence of Ni-ZIF-8@GOD&HRP, (C) Time-dependent curves for different concentrations of glucose (Glu) solutions, (D) Michaelis-Menten kinetics of the catalytic reaction with 20 mM H_2_O_2_ and 1.0 mM TMB under varying glucose concentrations catalyzed by Ni-ZIF-8@GOD&HRP, (E) Time-dependent curves for different concentrations of TMB solutions, (F) Michaelis-Menten kinetics of the catalytic reaction with 20 mM H_2_O_2_ and 1 mM glucose under varying TMB concentrations in the presence of Ni-ZIF-8@GOD&HRP.


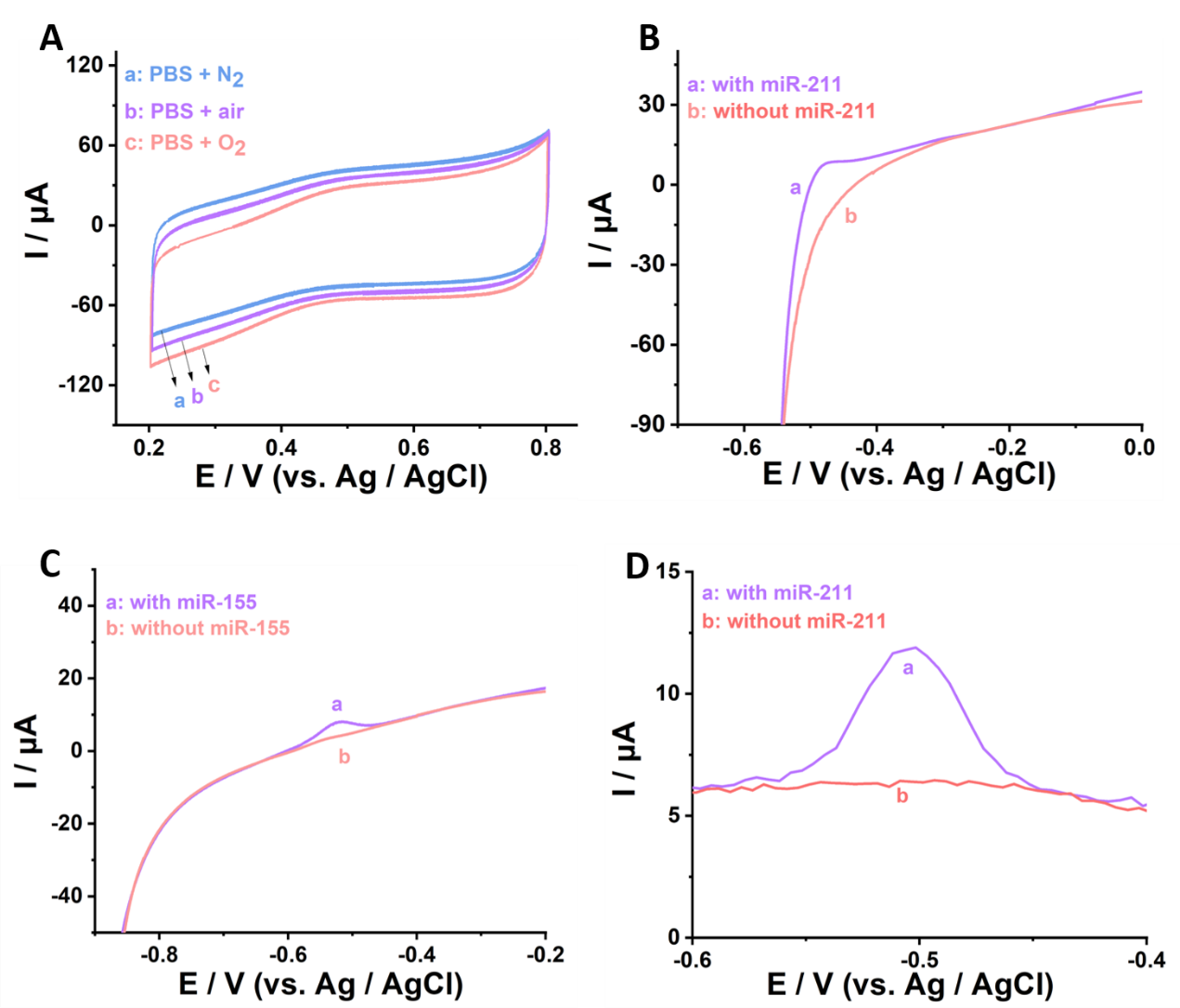


**Figure** S13. (A) Cyclic voltammetry (CV) curves of the biocathode in PBS solution under different gas environments: nitrogen (curve a), air (curve b), and oxygen (curve c), (B) Linear sweep voltammetry (LSV) curves of the bioanode in the presence of the target (a) and in the absence of miRNA-155 (b), (C) LSV curves of the bioanode in the presence of the target (a) and in the absence of miRNA-221 (b), (D) DPV curves of the bioanode in the presence of the target (a) and in the absence of miRNA-211 (b).


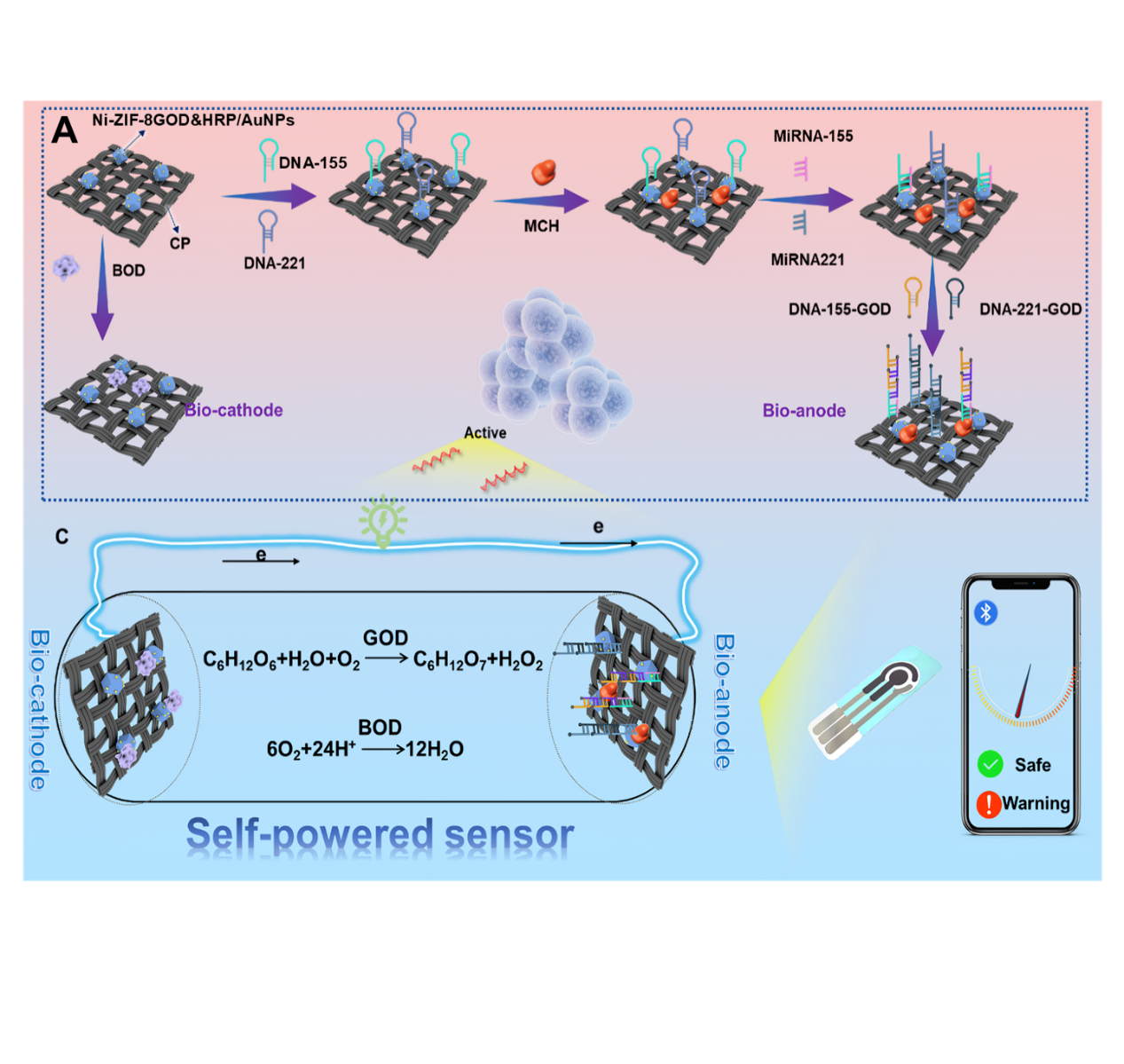

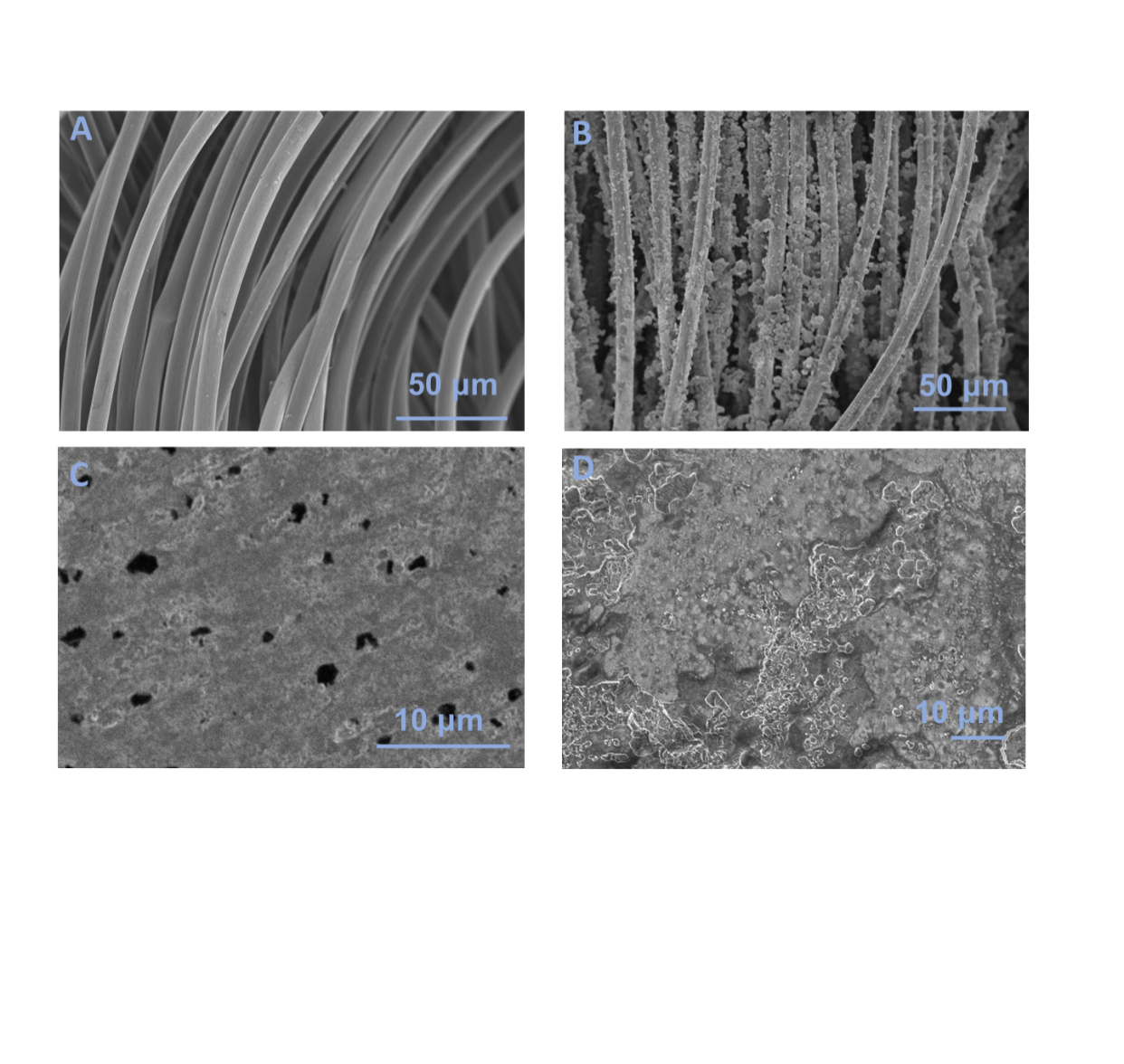
**Figure** S14. Microscopic images of the carbon cloth (A, B) and biosensor chip (C, D) before and after detection.

**Figure** S15. Schematic illustration of the construction and detection principle of the self-powered sensor based on a conjugate strategy.


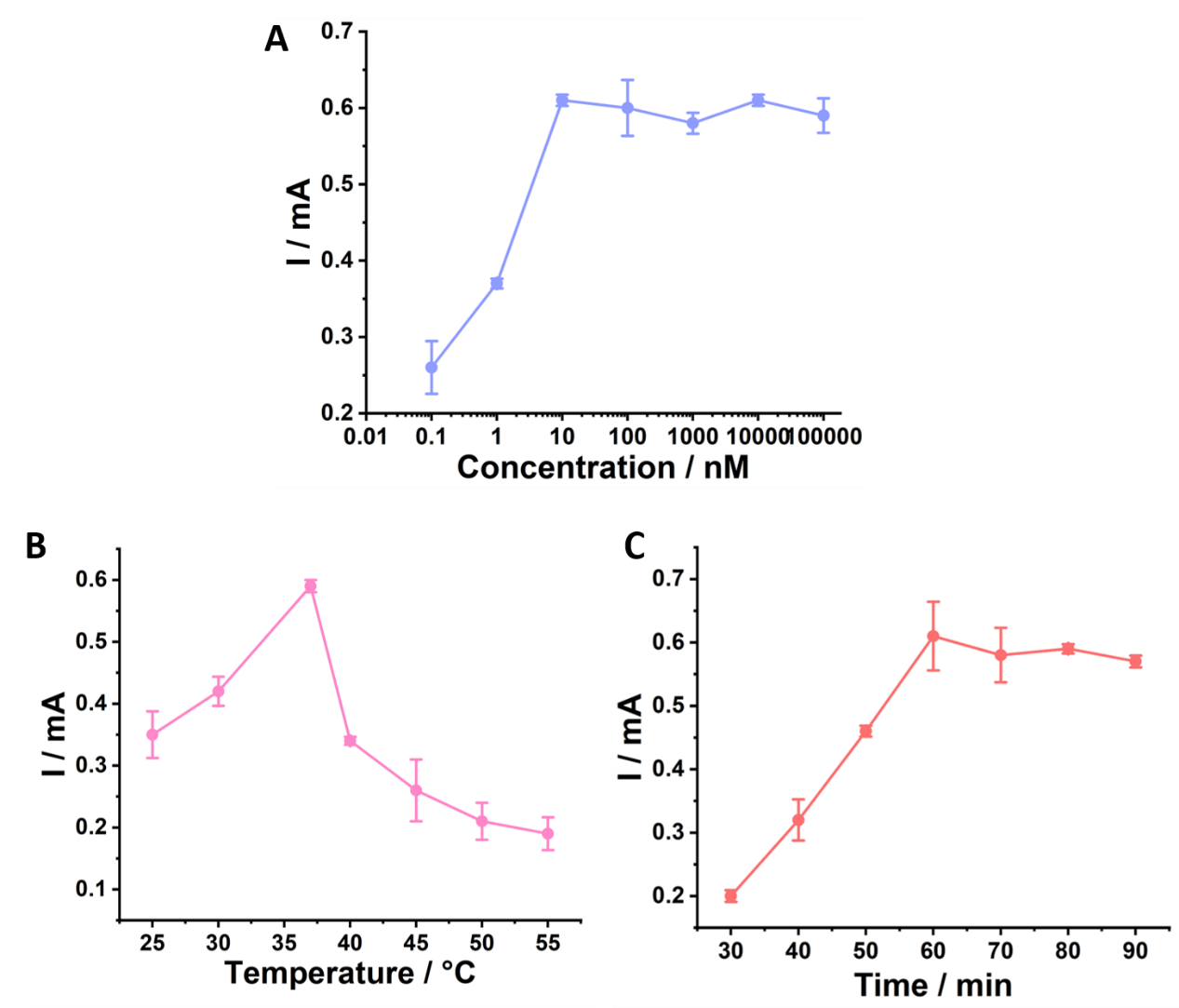


**Figure** S16. (A) Optimization of CP-DNA concentration. (B) Optimization of hybridization temperature. (C) Optimization of the binding time between CP-DNA-GOD and miRNA. The optimal conditions were determined to be 10 nM CP-DNA, 37 ℃, and 60 min, yielding the maximum current response.

**
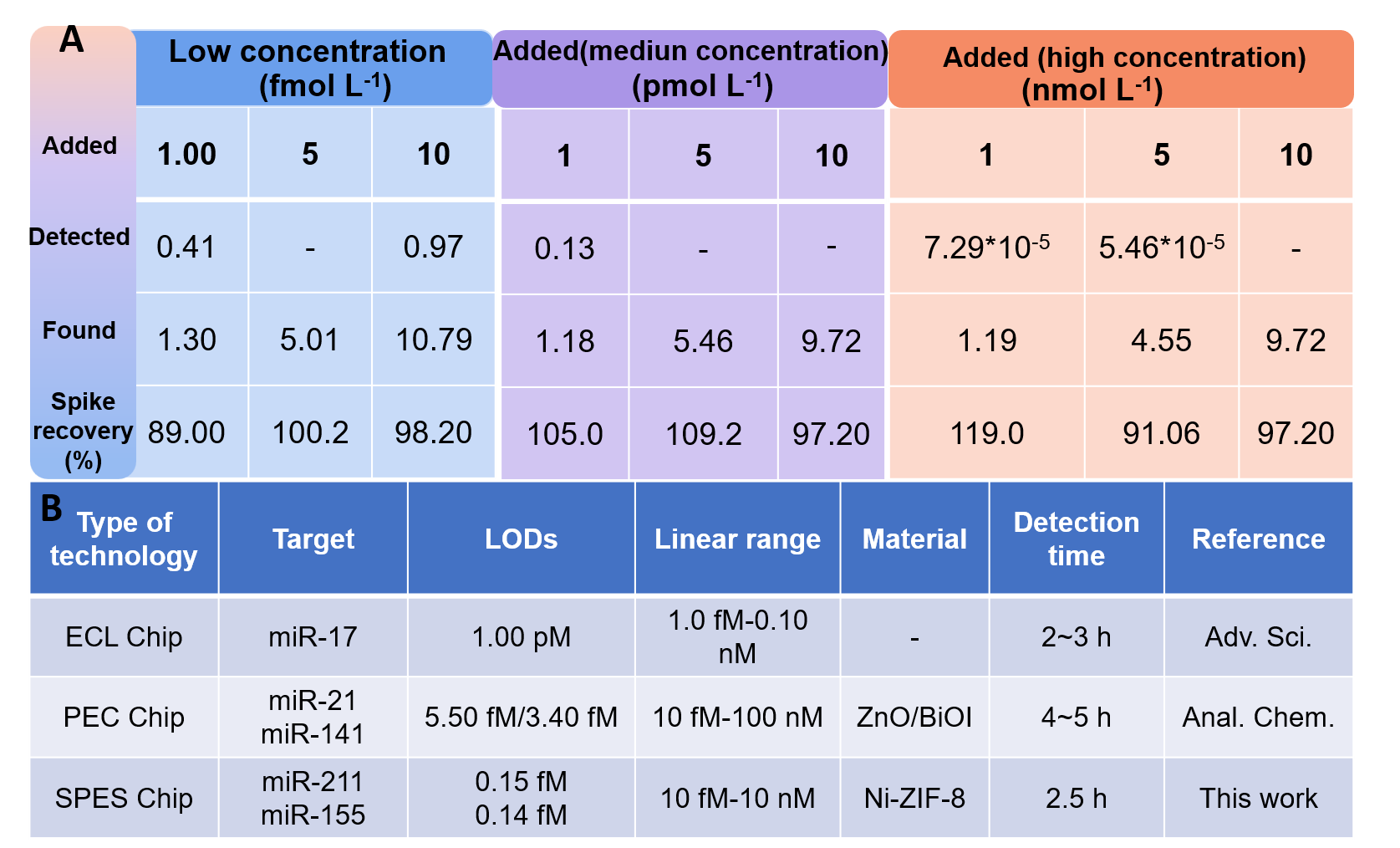
**

**Figure** S17. (A) Recovery results of miRNA-155 in different serum samples (n = 3), (B) Comparative analysis of the performance between the self-powered biosensor chip and other sensing platforms ^[2, 3]^.


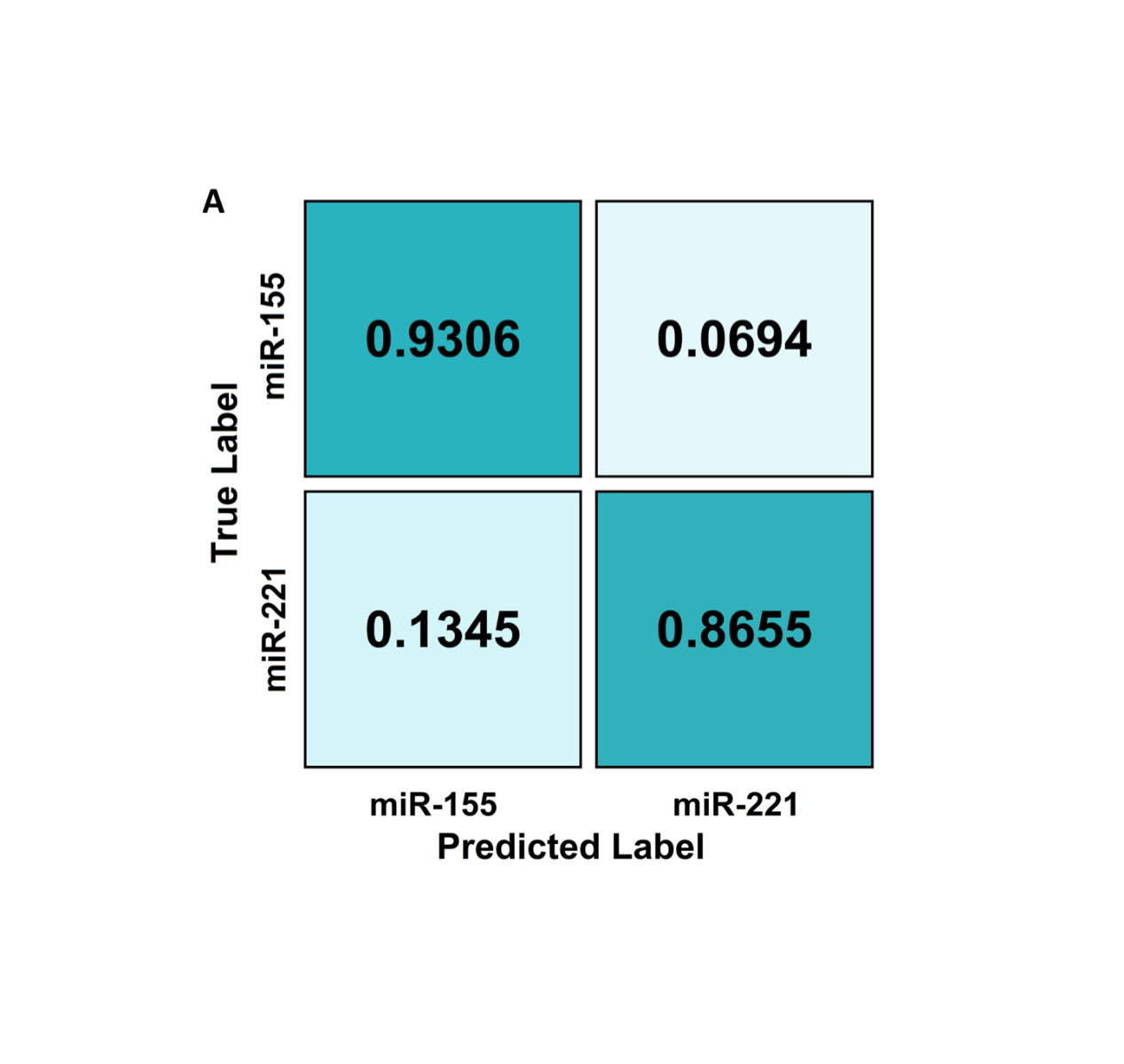
**Figure** S18. Confusion matrix illustrating the sample identification performance.


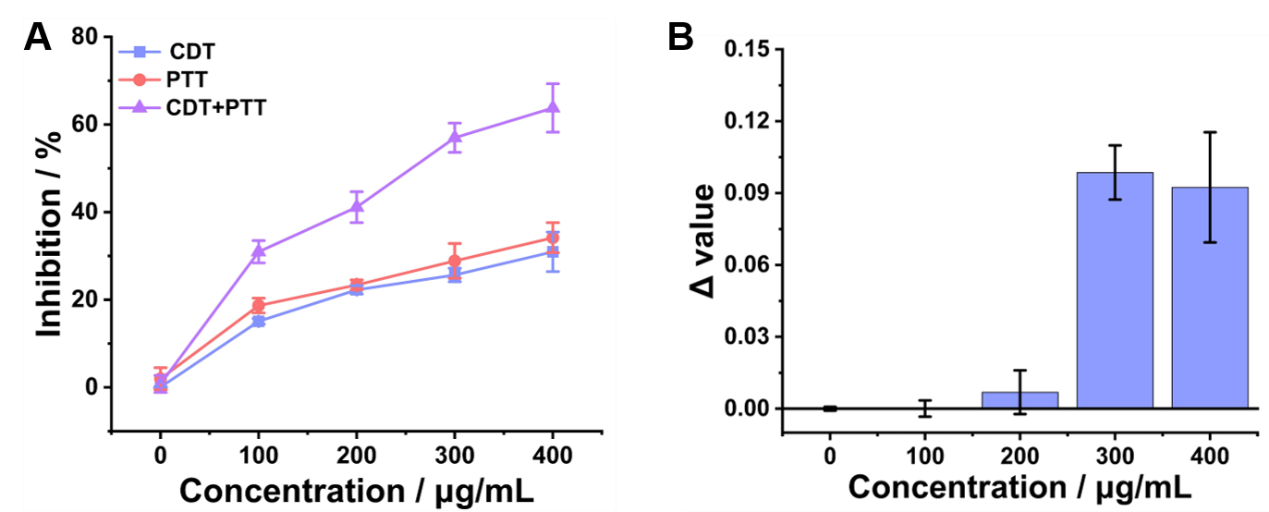


**Figure** S19. (A) Inhibition rates of CDT, PTT, and their combination (CDT+PTT) at different concentrations. (B) Quantitative evaluation of the synergistic effect using the Bliss Δ value. The positive Δ values at higher concentrations indicate a significant synergistic therapeutic effect between CDT and PTT.

**
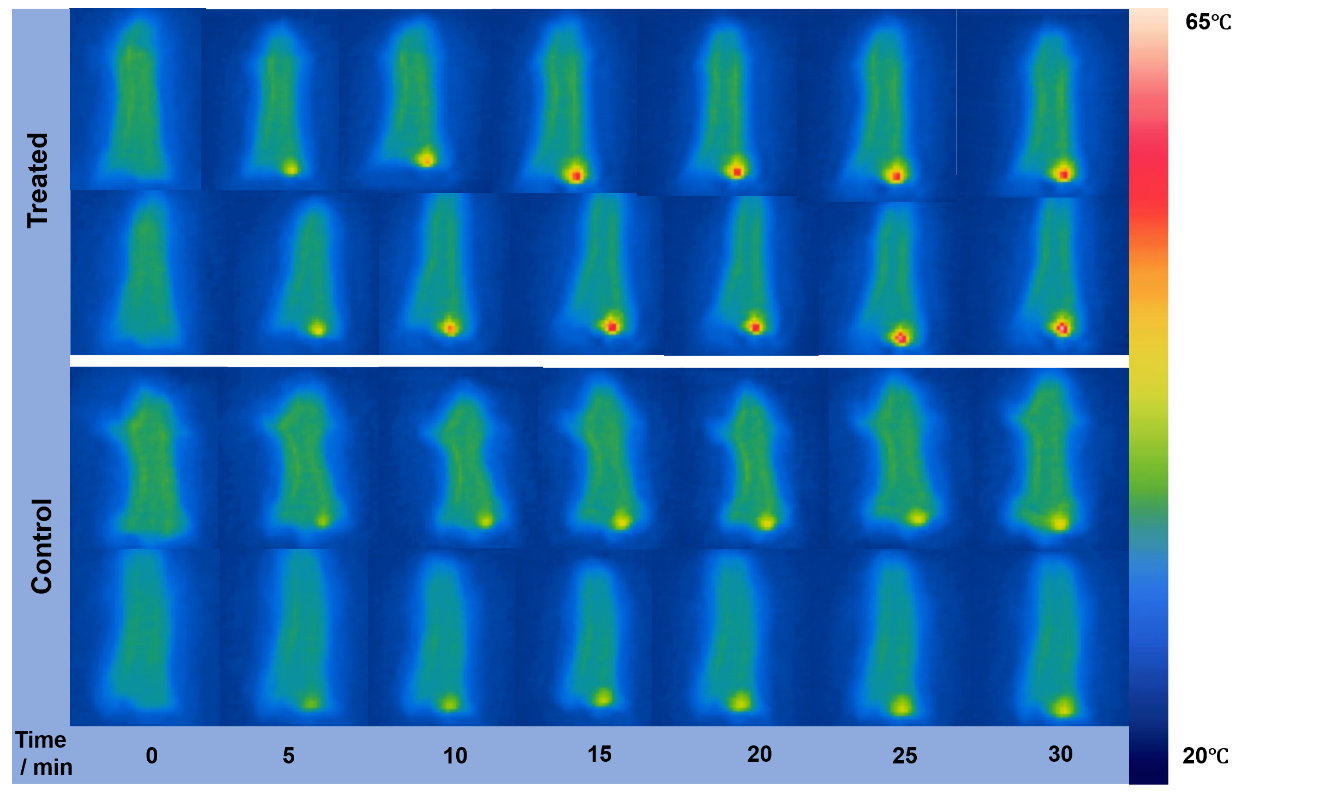
Figure** S20. Infrared thermal images showing the temperature changes of the experimental and control groups over time on days 7 and 14.


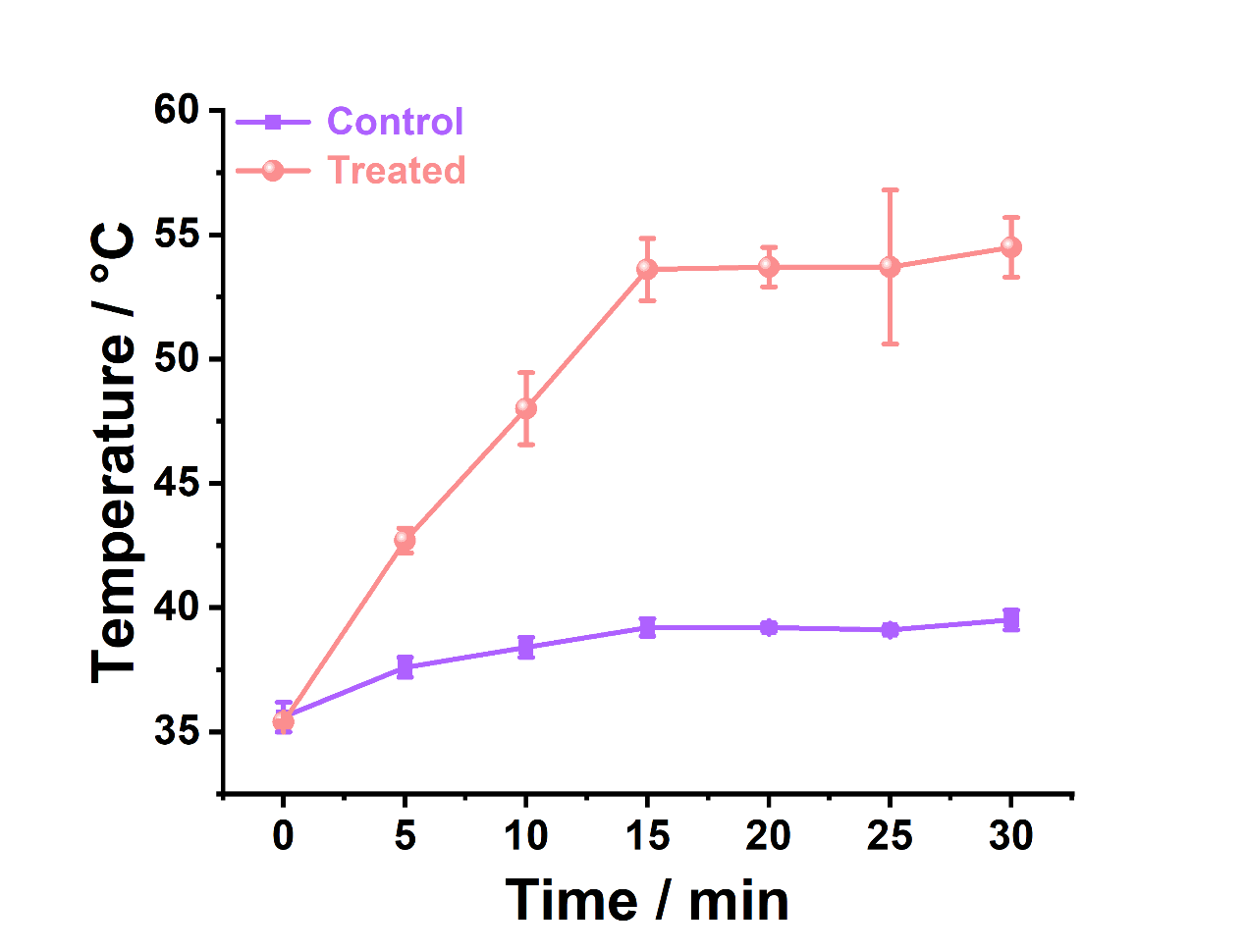


**Figure** S21. Temperature change curves over time for the experimental and control groups on days 7 and 14.


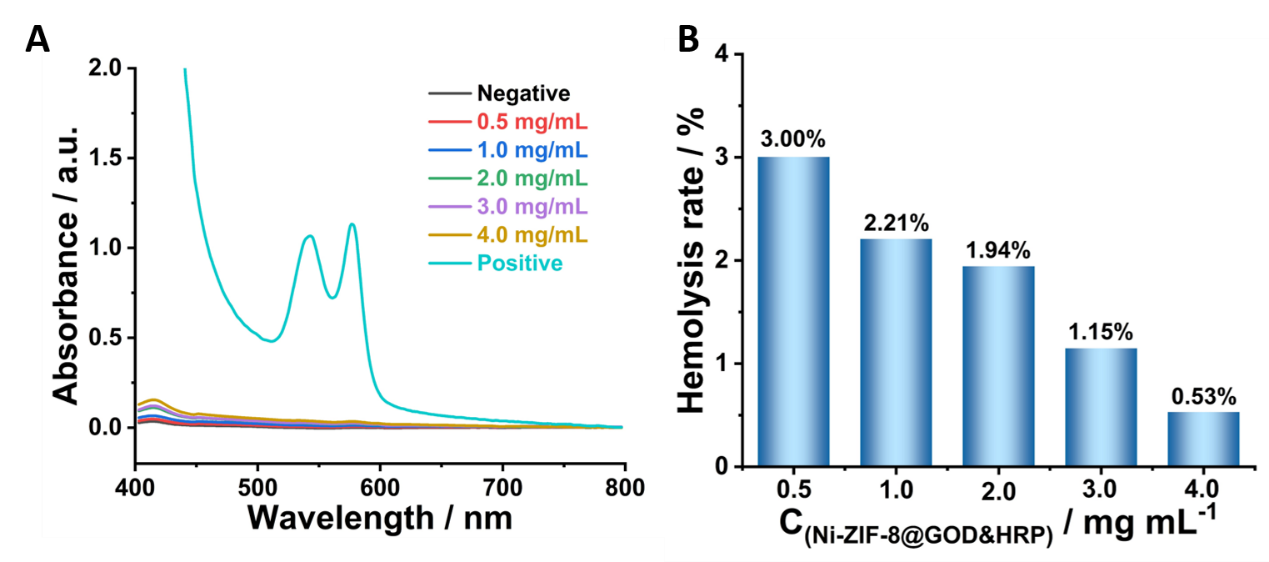


**Figure** S22. Hemolysis Assay: (A) Hemolysis analysis based on the UV-Vis absorption spectra of Ni-ZIF-8@GOD&HRP at different concentrations (by analyzing the absorbance of oxyhemoglobin (HbO_2_) at the β-band around 540 nm); (B) Bar chart representing the calculated hemolysis rates.

**Table S1.** DNA and RNA sequences used in the experiment.

| Name | Sequence |
| --- | --- |
| CP DNA-221 | ACA ATG TAG CT |
| CP DNA-155 | ACC CCT ATC AC |
| DNA-221 | SH-(CH_2_)_6_-TTT TTA ACG TAT GTC CTG CTG GGT TTC TTT TGA AAC CCA GCA G |
| DNA-155 | GAT TAG CAT TAA TTT TTT AAT GCT AAT ATC TGT CGA TCT TTT-(CH_2_)_6_-SH |
| miRNA-155 | UUA AUG CUA AUC GUG AUA GGG GU |
| miRNA-221 | AGC UAC AUU GUC UGC UGG GUU UC |
| miRNA-21 | UAG CUU AUC AGA CUG AUG UUG A |
| miRNA-20a | UAA AGU GCU UAU AGU GCA GGU AG |
| smRNA-221 | AGC UAC AUU GAC UGC UGG GUU UC |
| tmRNA-221 | AGC UUC AUU GUC UGG UGG GGU UC |
| NC-221 | UAG ACG UAG AGG ACU GCC AAG CG |
| smRNA-155 | UUA AUG CUA AUG GUG AUA GGG GU |
| tmRNA-155 | UUA AUGC CUA AUC GAG AUA GCG GU |
| NC-155 | AGU CAA GCU UAA CAC GGU CCA AC |

**Table S2.** DNA and RNA sequences used in the experiment.

| Catalyst | Substrate | *K_m_* (mM) | *V_max_* (10^-8^ M s^-1^) | Reference |
| --- | --- | --- | --- | --- |
| HRP | H_2_O_2_ | 3.70 | 8.71 | [4] |
|  | TMB | 0.43 | 10.00 |  |
| Fe_3_O_4_ | H_2_O_2_ | 154 | 9.7 | [5] |
|  | TMB | 0.098 | 3.44 |  |
| GO- Fe_3_O_4_ | H_2_O_2_ | 0.71 | 5.31 | [6] |
|  | TMB | 0.43 | 13.08 |  |
| Pd NSs | H_2_O_2_ | 4.39 | 6.51 | [7] |
|  | TMB | 0.11 | 5.82 |  |
| Ni-ZIF-8 @GOD&HRP | H_2_O_2_ | 3.50 | 78 | This  Work |
|  | Glucose | 0.72 | 117 |  |
|  | TMB | 2.18 | 333 |  |

**Table S3.** Ni content in Ni-ZIF-8@GOD&HRP nanoparticles determined by ICP-MS.

| **Sample** | **m_0_ (g)** | **C_0_ (mg/L)** | **Dilution**  **factor** | **C_1_ (mg/L)** | **Ni (wt%)** |
| --- | --- | --- | --- | --- | --- |
| 1 | 0.0520 | 2.1638 | 10 | 21.6384 | 1.0403% |
| 2 | 0.0520 | 2.1855 | 10 | 21.8549 | 1.0507% |
| 3 | 0.0520 | 2.1782 | 10 | 21.7822 | 1.0472% |
| Average ± SD | **-** | - | - | - | 1.046 ± 0.005 |

**References**

[1] Yang, X. G.; Zhang, J. R.; Tian, X. K.; Qin, J. H.; Zhang, X. Y.; Ma, L. F., Enhanced activity of enzyme immobilized on hydrophobic ZIF-8 modified by Ni^2+^ ions. Angewandte Chemie International Edition, 2023, 62 (7), e202216699.

[2] Zhou, T.; Huang, R.; Huang, M. Q.; Shen, J. J.; Shan, Y. Y.; Xin, D., CRISPR/Cas13a Powered Portable Electrochemiluminescence Chip for Ultrasensitive and Specific MiRNA Detection. Advanced. Science, 2020, 7, 1903661.
[3] Huang, C.; Zhang, L.; Zhu, Y. N.; Zhang, Z. H.; Liu, Y. Q.; Liu, C.; Ge, S. G.; Yu, J. H., Dual-Engine Powered Paper Photoelectrochemical Platform Based on 3D DNA Nanomachine-Mediated CRISPR/Cas12a for Detection of Multiple miRNAs. Analytical Chemistry, 2022, 94 (22), 8075-8084.
[4] Gao, L.; Zhuang, J.; Nie, L.; Zhang, J.; Zhang, Y.; Gu, N.; Wang, T.; Feng, J.; Yang, D.; Perrett, S.; Yan, X., Intrinsic peroxidase-like activity of ferromagnetic nanoparticles. Nature Nanotechnology, 2007, 2, 577-583.

[5] Ge, C. C.; Wu, R. F.; Chong, Y.; Fang, G.; Jiang, X. M.; Pan, Y.; Chen, C. Y.; Yin, J. J., Synthesis of Pt Hollow Nanodendrites with Enhanced Peroxidase-Like Activity against Bacterial Infections: Implication for Wound Healing. Advanced Functional Materials, 2018, 28, 1801484.

[6] Dong, Y. L.; Zhang, H. G.; Rahman, Z. U.; Su, L.; Chen, X. J.; Hu, J.; Chen, X. G., Graphene oxide-Fe_3_O_4_ magnetic nanocomposites with peroxidase-like activity for colorimetric detection of glucose. Nanoscale, 2012, 4, 3969-3976.

[7] Wei, J.; Chen, X.; Shi, S.; Mo, S.; Zheng, N., An investigation of the mimetic enzyme activity of two-dimensional Pd-based nanostructures. Nanoscale, 2015, 7, 19018-19026.
